# 0D Perovskites: Unique Properties, Synthesis, and Their Applications

**DOI:** 10.1002/advs.202102689

**Published:** 2021-10-24

**Authors:** Siqi Sun, Min Lu, Xupeng Gao, Zhifeng Shi, Xue Bai, William W. Yu, Yu Zhang

**Affiliations:** ^1^ State Key Laboratory of Integrated Optoelectronics and College of Electronic Science and Engineering Jilin University Changchun 130012 China; ^2^ Key Laboratory of Materials Physics of Ministry of Education School of Physics and Microelectronics Zhengzhou University Daxue Road 75 Zhengzhou 450052 China; ^3^ Department of Chemistry and Physics Louisiana State University Shreveport LA 71115 USA

**Keywords:** 0D structure, applications, optoelectronic properties, perovskites, synthetic methods

## Abstract

0D perovskites have gained much attention in recent years due to their fascinating properties derived from their peculiar structure with isolated metal halide octahedra or metal halide clusters. However, the systematic discussion on the crystal and electronic structure of 0D perovskites to further understand their photophysical characteristics and the comprehensive overview of 0D perovskites for their further applications are still lacking. In this review, the unique crystal and electronic structure of 0D perovskites and their diverse properties are comprehensively analyzed, including large bandgaps, high exciton binding energy, and largely Stokes‐shifted broadband emissions from self‐trapped excitons. Furthermore, the photoluminescence regulation are discussed. Then, the various synthetic methods for 0D perovskite single crystals, nanocrystals, and thin films are comprehensively summarized. Finally, the emerging applications of 0D perovskites to light‐emitting diodes, solar cells, detectors, and some others are illustrated, and the outlook on future research in the field is also provided.

## Graphical abstract

The unique crystal and electronic structure of 0D perovskites and their photoluminescence mechanism are comprehensively analyzed in this review. The different synthetic methods for 0D perovskite crystals and thin films, and their diverse applications are also discussed. In addition, the outlook on future research in the field is provided.

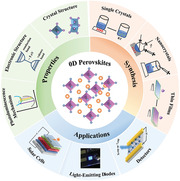

## 1 Introduction

Perovskite materials have gained much attention due to their high photoluminescence quantum yields (PL QY), high color purity, tunable bandgap, wide color gamut, high charge‐carrier mobilities, and long carrier diffusion lengths.^[^ ^1^ ^]^ These excellent optoelectronic properties make them widely applied in various optoelectronic devices.^[^ ^2^ ^]^ In just ten years, the power conversion efficiencies (PCEs) of perovskite solar cells have increased from 4%^[^ ^3^ ^]^ to 25.5%,^[^ ^4^ ^]^ which is comparable to that of crystalline silicon solar cells. In addition, perovskites have been widely used for light‐emitting diodes (LEDs) as emissive layer,^[^ ^5^ ^]^ and the external quantum efficiency (EQE) of perovskite LEDs raised rapidly from 0.1% in 2014^[^ ^6^ ^]^ to over 20%.^[^ ^7^ ^]^ Recently, perovskites are also proven to be promising candidates for other optoelectronic applications, including photodetectors,^[^ ^8^ ^]^ X‐ray detectors,^[^ ^9^ ^]^ and lasers.^[^ ^10^ ^]^

**Figure 1:**
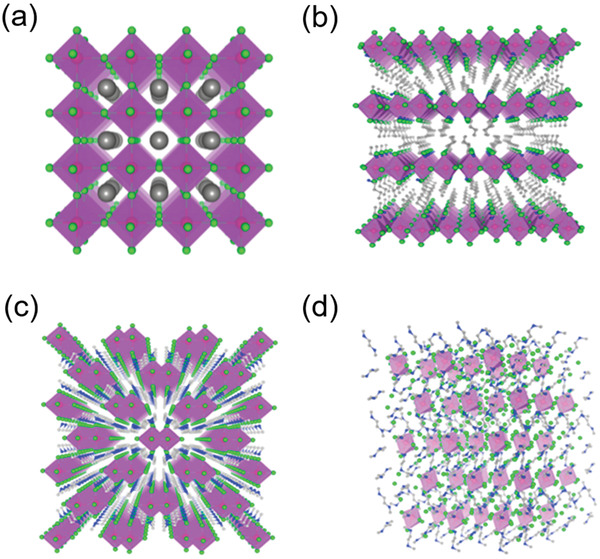
Crystal structures of a) cubic 3D perovskite, b) 2D perovskite, c) 1D perovskite, and d) 0D perovskite at molecular levels. Reproduced with permission.^[^ ^22^ ^]^ Copyright 2015, Elsevier.

By controlling appropriate organic and inorganic components to regulate structure dimensionality of perovskites, 3D, 2D, 1D, and 0D perovskites can be prepared (**Figure** **1**). 3D perovskites with general formula ABX_3_ (where A is monovalent organic or inorganic cation, B is divalent cation, and X is halide anion), consist of continuous corner‐sharing metal halide [BX_6_]^4−^ octahedra. The outstanding photovoltaic characteristics of 3D perovskites, such as long carrier diffusion length^[^ ^11^ ^]^ and low exciton binding energy,^[^ ^12^ ^]^ make them are primarily favored by researchers.^[^ ^13^ ^]^ However, these 3D perovskites suffer from phase transformation and instability, which hinders their practical application in the atmospheric environment. To overcome these limitations of 3D perovskites, low‐dimensional perovskites have been developed.^[^ ^14^ ^]^ By slicing along (100) or (110) crystallographic planes, (quasi‐) 2D or corrugated‐2D perovskites can be derived from 3D perovskites. And 1D and 0D perovskites can be obtained by further slicing 2D perovskites into metal halide wires and individual octahedra. Strong interactions between metal halide octahedra in both (quasi‐) 2D and 3D perovskites lead to the formation of electronic bands with small Stokes‐shifted narrow band emissions from free excitons (FEs).^[1f,^ ^15^ ^]^ Different from (quasi‐) 2D and 3D perovskites with narrow band emissions, corrugated‐2D and 1D perovskites, with simultaneous electronic bands formation and structural distortions, have large Stokes‐shifted broadband emissions from both FEs and self‐trapped excited states (STEs).^[^ ^15^, ^16^ ^]^ Despite the excellent stability of 2D and 1D perovskites is conducive to optoelectronic applications, the relatively low PL QYs limit their further commercialization.^[^ ^17^ ^]^ Particularly, 0D perovskites are composed of isolated metal halide octahedral anions or metal halide clusters that are separated from each other by surrounding inorganic or organic cations without the electronic bands formation. Therefore, the 0D perovskites retain the photophysical properties of individual metal halide octahedra or metal halide clusters and have strongly Stokes‐shifted broadband emissions with high PL QYs from STEs only.^[^ ^18^ ^]^ Especially, 0D Cs_4_PbBr_6_ perovskites exhibit narrow emissions, and their PL mechanism is still controversial. In addition, 0D perovskites have strong exciton binding energy that can increase the radiative recombination in optoelectronic devices.^[^ ^19^ ^]^ Moreover, 0D perovskites exhibit remarkable stability in the ambient, especially the 0D organic metal halide perovskites, because they can be considered as perfect host–guest systems with metal halide octahedron units/clusters protected by the organic shells. The property comparisons of 3D, (quasi‐) 2D, corrugated‐2D, 1D, and 0D perovskites (except Cs_4_PbBr_6_) are listed in **Table** **1**. Compared with the 3D, 2D, and 1D perovskites, the 0D perovskites exhibit the highest stability, which is more beneficial to commercial applications.

**Table 1:** Property comparisons of 3D, (quasi‐) 2D, corrugated‐2D, 1D, and 0D perovskites (except Cs_4_PbBr_6_)

Parameter	3D	(Quasi‐) 2D	Corrugated‐2D	1D	0D(except Cs_4_PbBr_6_)
Stokes shift [nm]	10–20	10–20	40–200	100–200	100–350
FWHM [nm]	12–40	10–30	90–250	100–200	100–200
PL QY [%]	≈100	≈80	≈20	≈30	≈100
*E*_b_ [meV]	20–60	100–400	100–400	100–500	150–600
PL mechanism	FEs	FEs	FEs and STEs	FEs and STEs	STEs
Stability	Low	Medium	Medium	Medium	High

FWHM = full width at half maximum; *E*_b_ = exciton binding energy

In recent years, various applications have been developed by utilizing these intriguing properties of 0D perovskites. For example, the broadband emission and large Stokes shifts of 0D perovskites make them advantageous in down conversion white LED applications as phosphors.^[^ ^18^ ^]^ Besides, the unique isolated structure of 0D perovskites could suppress ion migration in solar cells^[^ ^20^ ^]^ and X‐ray detectors.^[^ ^21^ ^]^ However, the development of 0D perovskite‐based applications is still in its infancy. To accelerate the development of 0D perovskite‐based applications, it is important to understand the fundamental photophysical characteristics of 0D perovskites, which are largely determined by their unique crystal and electronic structure. Nevertheless, there is no review summarizing and discussing the crystal and electronic structure of 0D perovskites. And a comprehensive overview of 0D perovskites, including their properties, synthesis, and applications, is also still lacking for the further research on them.

In this review, we aim to provide a comprehensive overview in properties, synthesis, and applications of 0D perovskites. We first discuss the unique crystal structure and electronic structure of 0D perovskites, along with the intriguing properties derived from their unique isolated structure. We also discuss the PL mechanisms from the emission of STEs, and the regulation of PL properties for 0D perovskites. Particularly, the controversial PL mechanisms of 0D Cs_4_PbBr_6_ perovskites in recent years are also summarized and analyzed. Then the synthetic methods of 0D perovskites published in recent advances are summarized, including single‐crystal growth methods, colloidal synthesis methods, and thin films deposition. Thereafter, their applications in optoelectronic devices are introduced, including LEDs, solar cells, detectors, and some other applications. Finally, a brief conclusion and some perspectives on the further research of 0D perovskites are presented. We hope this review will bring more attention in the field of 0D perovskites and stimulate further research on materials’ preparation and their practical applications.

## 2 Properties of 0D Perovskites

### 2.1 Crystal Structure

**Figure 2:**
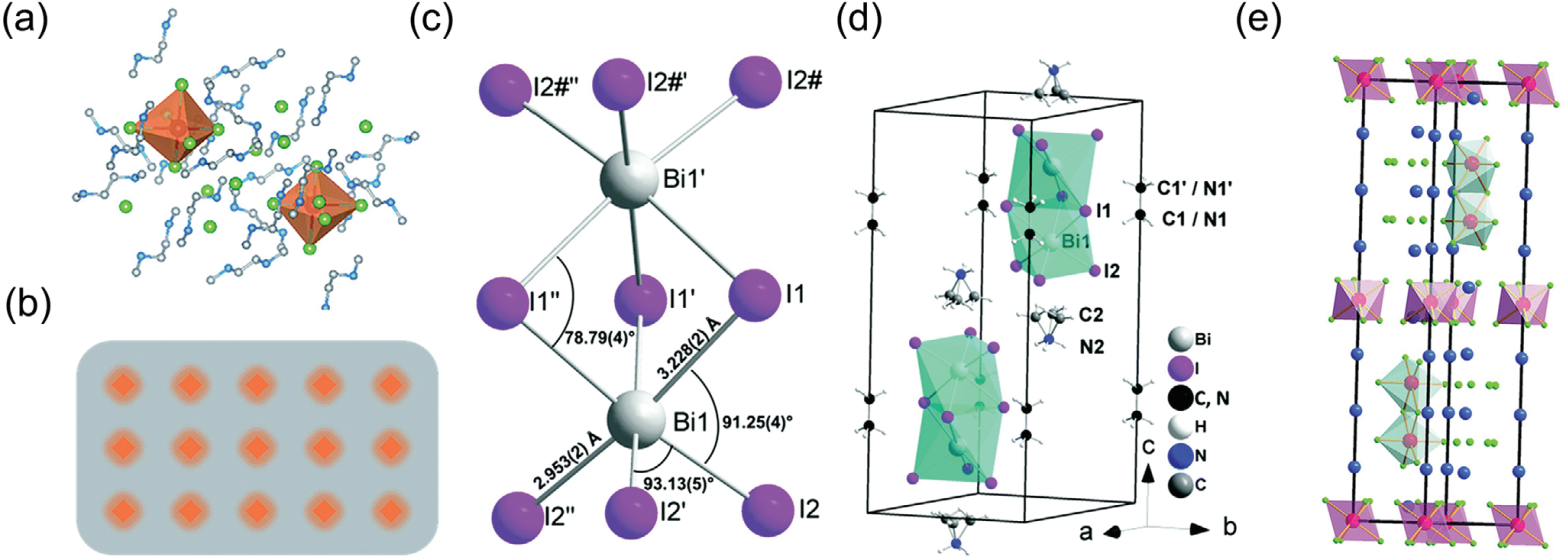
a) Views of two isolated [SnX_6_]^4−^ species completely separated from each other by large [C_4_N_2_H_14_Br]^+^ cations. b) Schematic drawing of a perfect host–guest system with the light emitting species periodically embedded in an inert matrix. a,b) Reproduced with permission.^[^ ^18^ ^]^ Copyright 2018, Royal Society of Chemistry. Crystal structure of (CH_3_NH_3_)_3_Bi_2_I_9_. c) Local structure of the [Bi_2_I_9_]^3−^ anion. d) Cation and anion positions in the unit cell. c,d) Reproduced with permission.^[^ ^25^ ^]^ Copyright 2016, Royal Society of Chemistry. e) Unit cell of Rb_7_Bi_3_Cl_16_ (pink octahedra represents the [BiCl_6_]^3−^ octahedra, blue edge‐sharing dimers represents the [Bi_2_Cl_10_]^4−^). Reproduced with permission.^[^ ^26^ ^]^ Copyright 2019, Royal Society of Chemistry.

0D perovskites are featured with isolated metal halide octahedral anions surrounded by organic or inorganic cations, where excitons are strongly confined to each octahedron. In a sense, they can be regarded as the core–shell structure (octahedron with negative charges as core, organic/inorganic cations as shell). The earliest report on 0D perovskite structure is Cs_4_PbCl_6_, in which [PbCl_6_]^4−^ octahedra are spatially isolated by surrounding Cs^+^ cations.^[^ ^23^ ^]^ But there are still strong interactions between [PbCl_6_]^4−^ octahedra, making the crystal structure of 0D Cs_4_PbCl_6_ similar to 3D structure. Recently, Ma's group^[^ ^18^ ^]^ replaced Cs^+^ cations with large organic cations and prepared bulk assemblies of 0D organic metal halide lead‐free (C_4_N_2_H_14_X)_4_SnX_6_ (X = Br, I) perovskites. In contrast to Cs_4_PbBr_6_, the (C_4_N_2_H_14_X)_4_SnX_6_ consists of isolated [SnX_6_]^4−^ species, which are completely separated from each other by large organic cations, with over a distance of 1 nm between two mental centers, as shown in **Figure** **2a**, thus leading to no interaction or electronic band formation between [SnX_6_]^4−^ species and further making the 0D (C_4_N_2_H_14_X)_4_SnX_6_ perovskites retain the intrinsic properties of isolated [SnX_6_]^4−^ species. Therefore, these 0D perovskites can be considered as perfect host−guest systems, in which metal halide octahedron units are periodically embedded in the wide‐bandgap matrix (Figure 2b).

Except for metal halide octahedra, 0D perovskites can also be composed of metal halide clusters. Jakubas's group first reported the (CH_3_NH_3_)_3_Bi_2_I_9_ single crystals, which is made up of isolated bioctahedral [Bi_2_I_9_]^3−^ anions and almost freely rotating CH_3_NH_3_^+^ cations. But Weissenberg photographs used in their study only allowed to suggest the space group *P6_3_/mmc*.^[^ ^24^ ^]^ Kaskel's group elucidated the correct crystal structure of 0D (CH_3_NH_3_)_3_Bi_2_I_9_ perovskite for the first time.^[^ ^25^ ^]^ The (CH_3_NH_3_)_3_Bi_2_I_9_ crystallizes in hexagonal phase, belonging to space group *P6_3_/mmc*. In this structure, two Bi atoms located on different mirror planes are bonded to three equivalent symmetrical bridging I1 atoms and terminal I2 atoms to form [Bi_2_I_9_]^3−^ complex anion, as shown in Figure 2c, showing slightly distorted octahedral coordination geometry. Thus the 0D (CH_3_NH_3_)_3_Bi_2_I_9_ consists of isolated face‐sharing [Bi_2_I_9_]^3−^ clusters that are separated by methylammonium cations, and the [Bi_2_I_9_]^3−^ anions are aligned along the *c* axis, corresponding crystal structure diagram is shown in Figure 2d. Similar to 0D (CH_3_NH_3_)_3_Bi_2_I_9_ perovskites, Xie et al.^[^ ^26^ ^]^ reported another 0D lead‐free Rb_7_Bi_3_Cl_16_ perovskites with cluster structure, which are composed of two kinds of octahedra with different octahedral distortions (Δd), discrete [BiCl_6_]^3−^ octahedra and [Bi_2_Cl_10_]^4−^ edge‐sharing dimers, and Rb^+^ cations filled in the space (Figure 2e). The [BiCl_6_]^3−^ octahedra, without any distortion (Δd1 = 0), are located on the surface of unit cell. The [Bi_2_Cl_10_]^4−^ dimers are located at the interior with larger distortions (Δd2 = 0.84). More [BiCl_6_]^3−^ octahedra on the surface of Rb_7_Bi_3_Cl_16_ are beneficial for its moisture stability.

### 2.2 Electronic Structure

For typical CsPbX_3_ 3D perovskite, the conduction band minimum (CBM) is derived from the Pb 6p orbits and halogen np orbitals, and the valence band maximum (VBM) is derived from the Pb 6s and halogen np orbitals.^[^ ^27^ ^]^ Therefore, the 3D structure of corner‐sharing [PbX_6_]^4−^ octahedra shows large band dispersion and low exciton binding energy, leading to easy exciton dissociation.^[^ ^28^ ^]^ While the generated excitons in the 0D perovskite can be confined in the isolated octahedra, thus making them unlikely dissociate and diffuse in the crystal lattice.^[^ ^29^ ^]^ Therefore, the 0D perovskites with isolated octahedral structure exhibit much larger bandgaps, stronger exciton binding energy and much smaller band dispersions than 3D perovskites.^[^ ^30^ ^]^ The bandgaps (*E*_g_) and exciton binding energy (*E*_b_) of 0D perovskites are summarized in **Table** **2**. In the electronic band structures of perovskites, the curvature of bands is relevant with charge carrier effective mass that directly affects the carrier mobility. Compared with the small charge carrier effective mass of 3D perovskites provided by the large band dispersion,^[^ ^31^ ^]^ 0D perovskites with flat CBM and VBM show much larger charge carrier effective mass, which means low carrier mobility.^[^ ^30^, ^32^ ^]^ The B—X—B bond angles in [BX_6_]^4−^ octahedra have been confirmed to have a strong influence on the band curvature, and thus influence the effective carrier masses and mobility. Lehner et al. reported that the near 180° B—X—B angles connecting the octahedra in the 3D perovskite structures facilitated a better orbital overlap, thus resulting in better carrier transport through the metal halide lattice than in the 0D perovskites A_3_Bi_2_I_9_ with Bi—I—Bi angles of around 150°.^[^ ^32b^ ^]^ Therefore, designing the structure of 0D perovskites with large B—X—B angles is a significant stagey to improve the carrier mobility for further application.

**Table 2:** Summary of bandgap (*E*_g_) and exciton binding energy (*E*_b_) for 0D perovskites

Perovskite material	Bandgap *E*_g_[eV]	Exciton binding energy *E*_b_ [meV]
Cs_4_PbBr_6_	3.95,^[^ ^42^ ^]^ 3.90,^[^ ^43^ ^]^ 4.80^[^ ^33^ ^]^	353,^[^ ^28a^ ^]^ 171,^[^ ^29^ ^]^ 159^[^ ^40a^ ^]^
Cs_4_PbI_6_	3.38,^[^ ^42a^ ^]^ 3.43^[^ ^43^ ^]^	
Cs_4_PbCl_6_	4.37,^[^ ^42a^ ^]^ 4.26^[^ ^43^ ^]^	
Cs_4_SnBr_6_	3.37,^[^ ^43^ ^]^ 3.33,^[^ ^34^ ^]^ 3.265,^[^ ^44^ ^]^ 5.01^[^ ^45^ ^]^	1205^[^ ^45^ ^]^
Cs_4_SnI_6_	3.03,^[^ ^43^ ^]^ 3.00^[^ ^34^ ^]^	
Cs_4_SnCl_6_	3.65^[^ ^43^ ^]^	
Cs_4_EuBr_6_	2.74^[^ ^35^ ^]^	
Cs_4_EuI_6_	2.70^[^ ^35^ ^]^	
Cs_3_Bi_2_I_9_	2.32,^[32b]^ 2.06^[^ ^46^ ^]^ 1.80,^[^ ^47^ ^]^ 2.86,^[^ ^39^ ^]^ 2.12^[^ ^36^ ^]^	300^[^ ^39^ ^]^
Cs_3_Sb_2_I_9_	2.40^[^ ^48^ ^]^	
Cs_3_Cu_2_I_5_	3.40^[^ ^49^ ^]^	490,^[^ ^41^ ^]^ 409^[^ ^49^ ^]^
Cs_2_SnCl_6_	3.95^[^ ^50^ ^]^	
CsSbBr_6_	1.85^[^ ^51^ ^]^	
N‐EtPySbBr_6_	1.55^[^ ^51^ ^]^	
N‐EtPySbCl_6_	1.65^[^ ^51^ ^]^	
(C_4_N_2_H_14_X)_4_SnBr_6_	5.10^[^ ^33^ ^]^	
(CH_3_NH_3_)_3_Sb_2_I_9_	2.19,^[^ ^52^ ^]^ 2.20^[^ ^53^ ^]^	
(CH_3_NH_3_)_3_Bi_2_I_9_	2.80,^[^ ^40b^ ^]^ 1.94,^[^ ^25^ ^]^ 2.1,^[^ ^54^ ^]^ 2.9,^[^ ^55^ ^]^ 2.26^[^ ^56^ ^]^	300,^[^ ^40b^ ^]^ 270,^[^ ^54^ ^]^ 260^[^ ^56^ ^]^
BA_3_Bi_2_I_9_	2.34^[^ ^56^ ^]^	130^[^ ^56^ ^]^
(CH_3_NH_3_)_4_PbI_6_·2H_2_O	3.87^[^ ^57^ ^]^	545^[^ ^57^ ^]^
(C_8_NH_12_)_4_Bi_0.57_Sb_0.43_Br_7_·H_2_O	2.84^[^ ^58^ ^]^	

**Figure 3:**
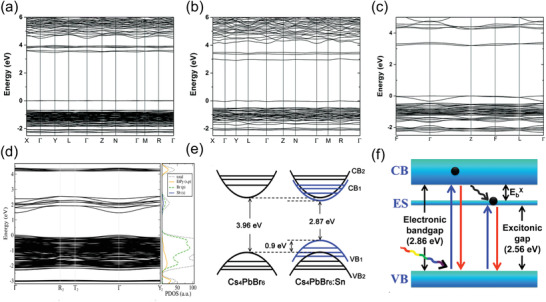
Electronic band structures of a) (C_4_N_2_H_14_Br)_4_SnBr_6_, b) (C_4_N_2_H_14_I)_4_SnI_6_, and c) Cs_4_PbBr_6_ calculated by Perdew–Burke–Ernzerhof (PBE) functionals. a‐c) Reproduced with permission.^[^ ^33^ ^]^ Copyright 2018, Royal Society of Chemistry. d) DFT‐calculated electronic band structure of EtPySbBr_6_ (left). Total state density of EtPySbBr_6_ and state density projected onto atomic orbitals (PDOS) of EtPy (s‐ and p‐states), Sb (s‐states), and Br (p‐states), showing Sb–Br hybridization and formation of intermediate bands (right). Reproduced with permission.^[^ ^37^ ^]^ Copyright 2018, American Chemical Society. e) Schematic illustration of the possible electronic dual‐bandgap structure for the as‐synthesized 0D Cs_4_PbBr_6_ perovskite NCs before and after Sn cation doping. Reproduced with permission.^[^ ^38^ ^]^ Copyright 2019, Wiley‐VCH. f) Schematic diagram showing the probable dual PL mechanism of 0D Cs_3_Bi_2_I_9_ perovskite NCs. Reproduced with permission.^[^ ^39^ ^]^ Copyright 2018, American Chemical Society.

**Figure** **3a**–c shows the electronic band structures of 0D (C_4_N_2_H_14_Br)_4_SnBr_6_, (C_4_N_2_H_14_I)_4_SnI_6_, and Cs_4_PbBr_6_, all of which valence band and conduction band are dominated by the electronic states originated from anionic [BX_6_]^4−^ octahedra.^[^ ^33^ ^]^ In addition, they all have the same formula A_4_BX_6_ (where A is monovalent organic or inorganic cation, B is divalent cation, and X is halide anion). The bandgaps of (C_4_N_2_H_14_X)_4_SnBr_6_, (C_4_N_2_H_14_I)_4_SnI_6_, and Cs_4_PbBr_6_ are 5.10, 4.43, and 4.80 eV, respectively. The large organic cations in the (C_4_N_2_H_14_X)_4_SnX_6_ (X = Br, I) can produce negligible electronic coupling between [SnX_6_]^4−^ octahedra, resulting in nearly flat conduction band and valence band. However, more conspicuous band dispersion is observed in Cs_4_PbBr_6_ due to the short intercluster Br–Br distance caused by the relatively small Cs^+^ cations, which illustrates that the A‐site cations can affect the electronic band structures of 0D perovskites. Based on the density functional theory (DFT) calculations, the band structures of Cs_4_SnBr_6_ and Cs_4_SnI_6_ both have a flat band edge, indicating the strong exciton confinement in the Sn‐based 0D perovskites. The bandgaps of Cs_4_SnBr_6_ and Cs_4_SnI_6_ were calculated to be 3.33 and 3.00 eV, respectively.^[^ ^34^ ^]^ The bandgaps are also affected by B‐site metal ions. Different from the Cs_4_SnX_6_ (X = Br, I), of which the CBM is mainly derived from Sn‐5p and the VBM is mostly composed of Sn‐5s and Br‐4p (or I‐5p) orbitals, respectively,^[^ ^34^ ^]^ Wu et al. reported the band structures of Cs_4_EuX_6_ (X = Br, I) that shows Eu‐4f‐derived VBM and the Cs‐6s‐derived CBM, both of which have no relation with halogen np orbitals.^[^ ^35^ ^]^ The Cs_4_EuX_6_ (X = Br, I) exhibits flat valence bands due to strong localization of the Eu‐4f orbitals. The calculated bandgaps of Cs_4_EuBr_6_ and Cs_4_EuI_6_ are 3.9 and 4.2 eV, respectively.

Dimensionality reduction of perovskites from 3D to 0D typically would result in larger bandgaps due to reduced orbital overlap.^[^ ^30b,c^ ^]^ However, the relatively large band gap of 0D perovskites is an obstacle to fabricate highly efficient perovskite optoelectronic devices, even though they have an excellent stability. Hong et al. found that applying dual metal cations such as Cs_3_BiXI_9_ (X: trivalent atom) can effectively reduce the bandgaps of Cs_3_Bi_2_I_9_.^[^ ^36^ ^]^ Through the theoretical investigation, they found that In and Ga can reduce the bandgaps of Cs_3_Bi_2_I_9_, due to the smaller lattice constant and volume. In addition, the 0D halide perovskites with a smaller bandgap can be obtained by changing the B‐site ions to introduce the intermediate bands in band structure. Egger reported 0D EtPySbBr_6_ perovskites that have an intermediate band in the band structure, as shown in Figure 3d.^[^ ^37^ ^]^ The valence bands are closely spaced in the range of 2.3 eV, and the conduction bands show two sets of bands with an energy gap about 1.8 eV between them, that is, eight dispersive bands and eight higher‐lying flat ones. This band structure is similar with the so‐called “intermediate bandgap material.” The formation of intermediate bands in EtPySbBr_6_ is mainly attributed to the orbital‐hybridization effects of the Sb s‐states. In addition, Zou et al. tailored the insulator bandgaps (≈3.96 eV) of Cs_4_PbBr_6_ to the visible blue spectral region by replacing the vast majority of the PbBr_2_ precursor with SnBr_2_ to form a Pb^2+^‐poor and Br^−^‐rich reaction environment.^[^ ^38^ ^]^ This method changed the local coordination environment of isolated [PbBr_6_]^4−^ octahedra in the Cs_4_PbBr_6_ crystal due to the Sn^2+^ cation doping. A new absorption peak at 432 nm was observed for the Sn‐doped 0D Cs_4_PbBr_6_ perovskite nanocrystals (NCs), indicating the existence of an additional electronic bandgap structure. Then based on the transient fs‐TA spectroscopy and first‐principles calculations, they confirmed the formation of an unusual electronic dual‐bandgap structure consisting of a new semiconducting bandgap of ≈2.87 eV and an original insulator bandgap of ≈3.96 eV. Furthermore, from the calculated densities of states (DOS), they found that the increased VBM for the Sn‐doped Cs_4_PbBr_6_ NCs is mainly attributed to the 5s‐orbitals of Sn^2+^, and the decreased CBM is predominately attributed to the synergistic effect of the 5s‐orbitals of Sn^4+^ caused by the oxidization of Sn^2+^, 6p‐orbitals of Pb^2+^, and 4s‐orbitals of Br^−^, which suggested that the coexistence of the Sn_Pb_ and Br_i_ point defects after Sn^2+^ cation doping would induce the unique electronic dual bandgap structure. (Figure 3e)

The isolated octahedra structure makes 0D perovskites exhibit stronger exciton binding energy than 3D perovskites. The strong exciton binding energy can effectively avoid the dissociation of excitons at room temperature and the formation of free charge carriers, which is beneficial to the realization of high‐performance optoelectronic devices that are based on photon emission.^[^ ^28^, ^29^, ^40^ ^]^ The exciton binding energies of 0D perovskites are about an order of magnitude larger than that of the 3D metal halide perovskites. Jun et al. reported the large exciton binding energy of 0D Cs_3_Cu_2_I_5_ perovskites to be 490 meV through measuring the PL intensity variation of Cs_3_Cu_2_I_5_ with temperature ranging from 30 to 350 K. The exciton binding energy can be calculated via this equation of $I\left(T\right)=\frac{I_{0}}{1+A\exp(-\frac{E_{b}}{k_{B}T})}\;$, where *E*_b_ is the exciton binding energy, *I*_0_ and *I*(*T*) are the integrated PL intensities when the temperatures are 0 and *T* K, respectively, and *k*_B_ is the Boltzmann constant.^[^ ^41^ ^]^ Pal et al. reported 0D Cs_3_Bi_2_I_9_ perovskite NCs with a large exciton binding energy of 300 meV.^[^ ^39^ ^]^ The energy of excitonic states (ES) is lower than the CBM, thus leading to two absorption features for electronic bandgap and excitonic gap. In addition, a blue shift and increased absorption peak intensity with the temperature decreasing from 296 to 10 K can be observed, which is attributed to exciton−phonon interactions. Moreover, they found the effective phonon energy of 36 meV is much smaller than the exciton binding energy, thus suppressing the rate of the phonon‐mediated relaxation from CBM to the ES, as shown in Figure 3f. Consequently, the CBM retains part of the excited electrons to undergo radiative recombination with the holes in valence band, while the other part of carriers emit light through the ES states, resulting in two PL peaks.

### 2.3 Photoluminescence Mechanism and Luminescence Regulation

**Figure 4:**
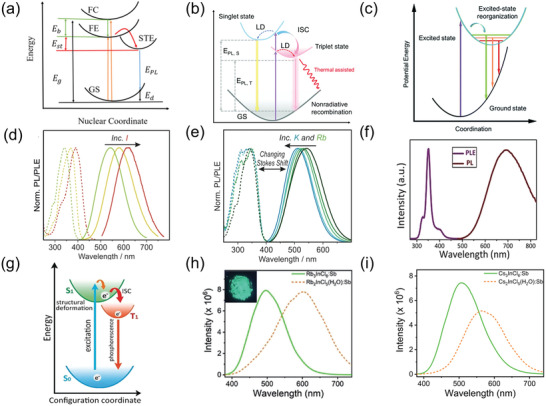
a) Schematic of typical STEs process, where GS represents ground state. FE represents free exciton state. FC represents free carrier state. STE represents self‐trapped exciton state. *E*_g_ represents bandgap energy, *E*_b_ represents exciton binding energy. *E*_st_ represents self‐trapping energy. *E*_d_ represents lattice deformation energy. *E*_PL_ represents emission energy. Reproduced with permission.^[^ ^63^ ^]^ Copyright 2019, American Chemical Society. b) Schematic of the luminescence processes in 0D perovskites, where LD represents lattice distortion. *E*_PL,S_ represents singlet state emission energy and *E*_PL,T_ represents triplet state emission energy. Reproduced with permission.^[^ ^65^ ^]^ Copyright 2021, The Royal Society of Chemistry. c) Schematic of excited state structural reorganization in (C_4_N_2_H_14_Br)_4_SnBr_6_ pure‐halide perovskites. Reproduced with permission.^[^ ^18^ ^]^ Copyright 2018, The Royal Society of Chemistry. d) PL and PL excitation (PLE) spectra of d) Cs_4_Sn(Br, I)_6_ and e) Rb^+^ or K^+^ substituted compounds. d,e) Reproduced with permission.^[^ ^70^ ^]^ Copyright 2018, Wiley‐VCH. f) Excitation (monitored at 695 nm) and emission (excited at 355 nm) spectra of a Cs_2_InBr_5_·H_2_O single crystal. g) Configurational coordinate diagram illustrating the origin of PL in Cs_2_InBr_5_·H_2_O. f,g) Reproduced with permission.^[^ ^74^ ^]^ Copyright 2019, Wiley‐VCH. h) Steady‐state PL spectra of Rb_2_InCl_5_ (H_2_O):Sb and Rb_3_InCl_6_:Sb, with the photograph of Rb_3_InCl_6_:Sb under UV light in the insets. i) The steady‐state PL spectra of Cs_3_InCl_6_:Sb and Cs_2_InCl_5_(H_2_O):Sb. h,i) Reproduced with permission.^[^ ^77^ ^]^ Copyright 2020, Wiley‐VCH.

The PL properties of most 0D perovskites are approximately determined by their completely isolated metal halide octahedra or clusters, resulting from the lack of electronic coupling between them.^[^ ^59^ ^]^ Different from 3D perovskites with small Stokes‐shift and narrowband emission, 0D perovskites generally show broadband emission with large Stokes shifts, which has been widely explained by STEs in low‐dimensional metal halide perovskites.^[^ ^22^, ^60^ ^]^ STE refers to the trapped bound exciton as a polaron in the distorted lattice field upon photoexcitation,^[^ ^33^ ^]^ which can easily occur in the perovskite materials with soft lattice and strong electron–phonon coupling.^[^ ^61^ ^]^ Therefore, many researchers have employed the STEs model to explain the largely Stokes‐shifted broadband emissions of 0D perovskites due to their easy octahedra distortion under photoactivation. **Figure** **4a** presents the process of typical STEs, in which free excitons are first formed by photogenerated free carriers and then relax into STEs, accompanied by the deformation of the surrounding lattice.^[^ ^62^ ^]^ And the large Stokes shift in STE emission originates from the collective effect of the energy loss of the exciton binding energy (*E*_b_), the self‐trapping energy (*E*_st_), and the lattice deformation energy (*E*_d_) in the process of STEs.^[^ ^63^ ^]^ However, this typical STEs mechanism encountered controversy in 0D perovskites. In contrast to corrugated 2D and 1D perovskites, of which the STE states need the free excitons to produce lattice distortion through the electron–phonon coupling,^[^ ^59^, ^62^, ^63^ ^]^ the photoexcited excitons in 0D perovskites will strongly distort the lattice of the excited state immediately after excitation and then will be localized to form STEs, without forming free excitons priorly. Because the extremely localization of excitons in the formation of 0D metal halide octahedra or clusters leads to the total disappearance of free excitons in 0D perovskites at room temperature.^[^ ^16^, ^62^, ^64^ ^]^ Totally, the STEs mechanism in 0D perovskites is present in Figure 4b, in which the excitons are excited to several excited states through the STEs process under excitations.^[^ ^65^ ^]^ Especially, the PL mechanism of 0D Cs_4_PbBr_6_ is highly controversial and will be discussed individually.

The isolated structure of 0D perovskites enables their strong quantum confinement effect, thus leading to higher PL QY in comparation to that of high‐dimensional perovskites. Sun et al. reported the highest efficient blue‐violet (392 nm) light emission of 0D [BAPrEDA]PbCl_6_·(H_2_O)_2_ with a PL QY of 21.3%, which far exceeds than that of the typical 3D CsPbCl_3_ (<5%).^[^ ^66^ ^]^ The enhancement of PL QY for the 0D perovskite was attributed to the strong quantum confinement effect of the 0D structure and highly localized electron in isolated [PbCl_6_]^4−^ octahedrons. Typical quantum dots with quantum confinement effect have been demonstrated to show markedly decreased PL QY when they are made into solid form,^[^ ^67^ ^]^ but the 0D perovskite Cs_4_PbBr_6_ solids still maintain high PL QY. Saidaminov et al. reported the 0D Cs_4_PbBr_6_ solids showed a PL QY of 45%, which was much higher than that of the 3D CsPbBr_3_ solids exhibiting ultralow luminescence (PL QY < 0.1%).^[^ ^28a^ ^]^ The high PL QY in 0D perovskites was due to their dramatically increased exciton binding energy that had been confirmed to be derived from their strong quantum confinement.^[^ ^66^ ^]^ Despite 0D perovskites exhibit excellent PL properties, PL regulation/optimization is still of vital importance for these materials to realize commercialization In general, the PL properties can be easily regulated by changing the chemical compositions and the synthetic temperature. Some representative researches on the PL regulation of 0D perovskites in the past several years are discussed below.

Sn^2+^ has been considered as one of the ideal substitutes for the toxic lead in metal halides due to its same lone‐pair s orbitals and similar ionic radius as Pb^2+^.^[^ ^68^ ^]^ Ma's group reported a broadband yellow emitting 0D tin‐based perovskite (C_4_N_2_H_14_Br)_4_SnBr_6_ with a peak emission at 570 nm, FWHM of 105 nm, large Stokes shift of 215 nm, and a near‐unity PL QY.^[^ ^18^ ^]^ The excited state processes for the 0D (C_4_N_2_H_14_Br)_4_SnBr_6_ perovskites are shown in Figure 4c. After absorbing the photon, the metal halide octahedron is firstly excited to a high‐energy excited state, and then transferred into a low‐energy excited state through an ultrafast excited state structural reorganization, and finally resulting in a broadband emission with large Stokes shift. This process is similar with the broadband emissions of corrugated‐2D and 1D metal halide perovskites that result from STEs.^[^ ^16^, ^62^ ^]^ Moreover, this 0D perovskite shows photostability under continuous irradiation and high thermal stability, resulting from the well protection of the photoactive metal halide octahedron by the organic shells. Subsequently, they reported another 0D tin mixed‐halide perovskite (C_4_N_2_H_14_Br)_4_SnBr_3_I_3_ by incorporating both bromide and iodide in the metal halide octahedron,^[^ ^69^ ^]^ which shows a yellow emission with FWHM of 126 nm from STEs. Compared to pure‐halide perovskites (C_4_N_2_H_14_Br)_4_SnX_6_ (X = Br, I) with one energy minimum resulting from the structural distortion of Sn–X, two or more energy minima are formed in the mixed‐halide perovskite caused by the structural distortion of either Sn–Br or Sn–I upon photoexcitation, leading to their much broader emissions than those of pure‐halide perovskites. In addition, excitons located in two energy minima could reach thermally activated equilibrium at room temperature, leading to the excitation‐independent PL of mixed‐halide perovskite. However, there is no thermally activated equilibrium at 77 K, and thus the overall emission spectrum is a combination of decays from different excitation‐dependent distorted structures, leading to the excitation‐dependent PL, which is not observed in 0D pure‐halide perovskites.

Kovalenko's group reported the broadband green–yellow emission of Cs_4_SnBr_6_ with a PL QY of 15% ± 5%.^[^ ^70^ ^]^ In addition, the Cs_4_SnBr_6_ exhibits a large Stokes shift of about 1.2 eV and long radiative lifetimes (540 ns at room temperature and 1381 ns at 200 K), together with the broadband emission, indicating the formation of STEs. Furthermore, the PL peak position and Stokes shifts of Cs_4_SnBr_6_ can be tuned through the partial substitution of both A‐site cations (Cs with Rb, K) and the halide anions (Br with I) to form the general formula Cs_4‐_ *_x_* A*_x_* Sn(Br_1‐_ *_y_* I*_y_*)_6_. As shown in Figure 4d, the photoluminescence excitation (PLE) and PL spectra show a red shift with the increase of I contents, and the PL peak at 620 nm was obtained when the ratio of Br:I is 1:1. In the case of A‐site cations substitution with K^+^ and Rb^+^ cations, the PL spectra show a blue shift, and the PLE spectra are almost unchanged, thus showing a reduced Stokes shift with K^+^ and Rb^+^ cations increasing (Figure 4e). Through the theoretical calculation, a pseudo‐Jahn–Teller distortion was observed in Cs_4_SnBr_6_ and decreased from Cs^+^ to Rb^+^ to K^+^, which enables a smaller Stokes shift and finally results in a blue shift in the PL spectra. Bi^3+^ with 6s^2^ lone‐pair states is also a widely studied alternative to the toxic Pb^2+^.^[^ ^71^ ^]^ McCall et al. reported the red emitting Cs_3_Bi_2_I_9_ with broadband emission.^[^ ^46^ ^]^ The Cs_3_Bi_2_I_9_ with a 0D molecular structure exhibits efficient self‐trapping behaviors due to the easy binding between the charged Cs^+^ cations and [Bi_2_I_9_]^3−^ clusters. Combining low‐temperature PL measurements and Raman spectroscopy, strong electron–phonon coupling in the 0D Cs_3_Bi_2_I_9_ perovskite was found. The electron–phonon interactions would induce polarons that are trap charge carriers, and thus produced STEs.

In‐based all‐inorganic metal halide perovskites have emerged as potential optoelectronic material due to their excellent stability and environmental friendliness.^[^ ^72^ ^]^ Moreover, In‐based all‐inorganic metal halide perovskites show better optoelectronic characteristics with decreased dimensions, but the related research is few, especially on 0D In‐based all‐inorganic perovskites.^[^ ^73^ ^]^ Zhou et al. reported a red emitting 0D In‐based perovskite single crystal (Cs_2_InBr_5_·H_2_O) with a PL QY of 33%.^[^ ^74^ ^]^ The Cs_2_InBr_5_·H_2_O shows a broadband emission peak at 695 nm and a sharp excitation peak at 355 nm (Figure 4f), thus producing a large Stokes shift of over 340 nm. Such a large Stokes shift indicates a negligible self‐absorption that is highly desirable for light‐emitting applications. In addition, this 0D perovskites have a prolonged lifetime (≈2.47 µs) at low temperatures relative to that of 1.67 µs at room temperature, suggesting a phosphorescent emission mechanism.^[^ ^75^ ^]^ The photophysical processes of Cs_2_InBr_5_·H_2_O are shown in Figure 4g. Electrons are excited from the highest occupied molecular orbital to the lowest occupied molecular orbital upon photoexcitation and simultaneously generated a structural deformation, which lead to the formation of STEs. The excited electrons tend to the lower triplet state (T1) from the singlet state (S1) through an intersystem crossing,^[^ ^76^ ^]^ and thus resulting in a broadband phosphorescence‐emission with a large Stokes shift. Afterward, Han et al. reported undoped and Sb‐doped all inorganic 0D perovskite single crystals A_2_InCl_5_ (H_2_O) (A = Rb, Cs) with enhanced broad yellow PL emission, resulting from the optimized bandgap structure and enhanced excitonic absorption caused by Sb^3+^ doping.^[^ ^77^ ^]^ Furthermore, Sb‐doped A_2_InCl_6_ (A = Rb, Cs) were also synthesized to investigate the influence of H_2_O on PL property. Compared to Sb‐doped A_2_InCl_6_ (A = Rb, Cs), Sb‐doped A_2_InCl_5_ (H_2_O) (A = Rb, Cs) shows red‐shifted PL emission from green to yellow, as shown in Figure 4h,i, resulting from the Jahn–Teller distortion induced by the In—O coordinate bond, which indicates that the PL emission color in 0D perovskites can be changed by controlling distortion of octahedron.

### 2.4 The Controversial Case of 0D Cs_4_PbBr_6_

**Figure 5:**
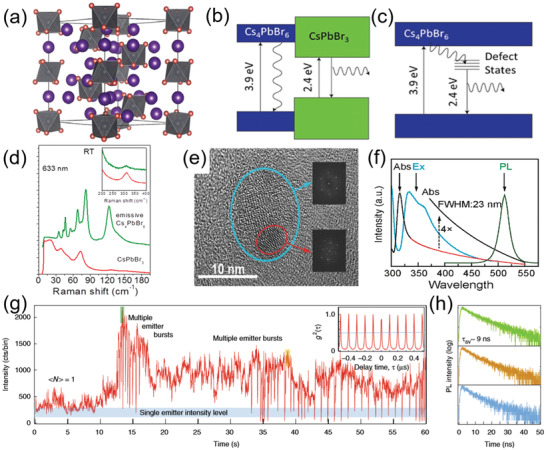
a) Crystal structure of Cs_4_PbBr_6_. Reproduced with permission.^[^ ^30a^ ^]^ Copyright 2017, American Association for the Advancement of Science. Luminance mechanism of Cs_4_PbBr_6_ from b) CsPbBr_3_ NC impurity phases and c) defect states. b,c) Reproduced with permission.^[^ ^22^ ^]^ Copyright 2015, Elsevier. d) Raman spectral comparison between Cs_4_PbBr_6_ and CsPbBr_3_ at room temperature. Reproduced with permission.^[^ ^83^ ^]^ Copyright 2019, American Chemical Society. e) HRTEM image of the Cs_4_PbBr_6_ crystals with embedded CsPbBr_3_ NCs. Reproduced with permission.^[^ ^84^ ^]^ Copyright 2018, Wiley‐VCH. f) Absorption, excitation, and PL spectra of Cs_4_PbBr_6_ NCs. Reproduced with permission.^[^ ^29^ ^]^ Copyright 2017, American Chemical Society. g) Blinking trace of a single Cs_4_PbBr_6_ NC, a typical antibunching trace with large *g* ^2^(0) value shown in the insets. h) PL lifetimes extracted from individual bursts (green and yellow) and single emitter level (blue), showing a similar decay profile. g,h) Reproduced with permission.^[^ ^86^ ^]^ Copyright 2019, Springer Nature.

Cs_4_PbBr_6_, a 0D perovskite with isolated [PbBr_6_]^4−^ octahedra separated by Cs^+^ ions (**Figure** **5a**), has gained broad attention of researchers due to their unprecedented PL QY in solid form, unique crystal structure, and optoelectronic properties.^[^ ^28^, ^78^ ^]^ However, the PL mechanism of Cs_4_PbBr_6_ is highly controversial. In the early 1990s, the anomalous light emission of Cs_4_PbBr_6_ was attributed to the inevitable CsPbBr_3_ phase during the single‐crystal growth.^[^ ^42b^ ^]^ In 2017, Zhang et al. reported the first luminous Cs_4_PbBr_6_ NCs with pure phase, which is attributed to the emission of defect states.^[^ ^29^ ^]^ While the nonemissive Cs_4_PbBr_6_ NCs were reported just one week later.^[^ ^42a^ ^]^ Therefore, there are mainly two views on the origin of green emission for Cs_4_PbBr_6_, that are CsPbBr_3_ inclusion/impurity emission and defect‐state emission. Figure 5b,c schematically shows these two PL mechanisms of Cs_4_PbBr_6_. It is noted that there is no energy transfer between CsPbBr_3_ and the band structure of Cs_4_PbBr_6_ in the PL mechanism originated from CsPbBr_3_ inclusion/impurity emission (Figure 5b). However, in Figure 5c showing defect‐state emission mechanism, there is an energy transfer process from the conduction band of the Cs_4_PbBr_6_ to the defect states that located in the sub‐bandgap.

There are many similar PL properties for CsPbBr_3_ and Cs_4_PbBr_6_, such as similar green emission peak at around 520 nm, narrow FWHM, small Stokes shift and high PL QY. The Cs_4_PbBr_6_ can be synthesized by regulating the molarity ratio of Cs/Pb and the oleylamine (OLA) ligands, but this process may cause inevitable existence of CsPbBr_3_.^[^ ^79^ ^]^ Therefore, many groups have attributed the PL emission of Cs_4_PbBr_6_ to CsPbBr_3_ inclusion^[^ ^80^ ^]^ or impurity,^[^ ^81^ ^]^ and there are many techniques to prove the existence of the CsPbBr_3_ phase. Song and co‐workers synthesized phase‐adjustable CsPbBr_3_@Cs_4_PbBr_6_ composite NCs and proved the existence of CsPbBr_3_ phase in the emissive Cs_4_PbBr_6_ by experimental X‐ray diffraction for the first time.^[^ ^82^ ^]^ Qin et al. used a cryo‐Raman spectroscopy technique to reveal the existence of CsPbBr_3_ in Cs_4_PbBr_6_ NCs (Figure 5d).^[^ ^83^ ^]^ Chen et al. reported centimeter‐sized Cs_4_PbBr_6_ crystals with superior PL. Figure 5e shows the transmission electron microscopy (TEM) images of small particles from green emissive crystals by ball milling technique. Obviously, many small‐sized CsPbBr_3_ partials were observed in Cs_4_PbBr_6_.^[^ ^84^ ^]^

However, the presence of CsPbBr_3_ will bring irreversible damage to the original PL emission of Cs_4_PbBr_6_, which is contrary to the hypothesis that the PL emission originates from CsPbBr_3_ phase.^[^ ^28^, ^40^, ^85^ ^]^ Zhang et al. combined experimental and theoretical research to further illustrate the luminous mechanism of Cs_4_PbBr_6_ NCs at single‐particle level.^[^ ^86^ ^]^ Through a blinking trace of a 0D perovskite Cs_4_PbBr_6_ at 〈*N*〉 (number of excitons) = 1 excitation level, they observed the appearance of multiple emitters in a single NC induced by an increased excitation power (Figure 5g). While the lifetimes extracted from individual bursts (green‐ and yellow‐colored boxes in the blinking trace) and from the single emitter level (blue shadowed bar) are very similar (Figure 5h), demonstrating the appearance of multiple emitters but not multiexcitons. Therefore, based on blinking traces and lifetime measurements mentioned above, they found the emission of Cs_4_PbBr_6_ NCs resembles that of the individual single molecule, rather than typical quantum‐confined semiconductors. In addition, the absorption spectrum of Cs_4_PbBr_6_ NCs shows a sharp peak at 315 nm and a long Urbach tail (Figure 5f), indicating the existence of defect states in Cs_4_PbBr_6_ lattice.^[^ ^29^, ^86^ ^]^ Furthermore, through the DFT calculation, the green PL emission in Cs_4_PbBr_6_ NCs was identified to the exciton recombination at Br vacancy sites within [PbBr_6_]^4−^ octahedra. While the chemical nature of the defect is still underexplored, thus resulting in various theories. For example, Hu et al. proposed that the voids among the isolated octahedra in 0D structure of Cs_4_PbBr_6_ may absorb small molecules like H_2_O and —OH groups. They further found that the —OH group can form a 2.6 eV sub‐bandgap in the band structure of Cs_4_PbBr_6_ based on the theoretical calculations, and this sub‐bandgap is consistent with the green emission energy.^[^ ^43^ ^]^ Jung et al. proposed that excess bromine will form deep defect levels, thus resulting in the formation of molecular Br_3_‐type species (Br_3_^−^, Br_3_^2−^, and Br_3_^3−^) that exhibits a range of optical transitions. Furthermore, they ascribed this defect‐mediated green PL to the fluorescence of optically excited Br_3_^−^ based on the theoretical calculations.^[^ ^87^ ^]^ In addition, many groups also reported that the green PL may be derived from STEs. Because the soft Cs_4_PbBr_6_ with isolated [PbBr_6_]^4−^ octahedra can be easily distorted under photoactivation, thus resulting in the formation of a STE or polaron.^[^ ^88^ ^]^ However, the views on STEs accounting for the green PL emission of Cs_4_PbBr_6_ may be illogical, because the typical features of STEs emission are broadband emission and long lifetime,^[^ ^63^ ^]^ which conflicts with the narrow PL emission of Cs_4_PbBr_6_.

## 3 Synthesis of 0D Perovskites

With the in‐depth study of 0D perovskite, various synthetic methods have been proposed. **Table** **3** summarized the synthetic methods of the 0D perovskites including single‐crystal growth methods, colloidal synthesis methods, and thin films deposition.

**Table 3:** Summary of the main synthesis methods for 0D perovskite

Perovskite single crystals	Synthesis method
(C_4_N_2_H_14_Br)_4_SnX_6_ (X = Br or I)	Antisolvent vapor crystallization,^[^ ^69^, ^89^ ^]^ solution growth^[^ ^18^ ^]^
(C_4_N_2_H_14_Br)_4_SnBr*_x_* I_6‐_ *_x_* (*x* = 3)	Antisolvent vapor crystallization^[^ ^70^ ^]^
(CH_3_NH_3_)_3_Bi_2_I_9_	Cooling‐induced crystallization,^[^ ^40b^ ^]^ seed‐crystal‐assisted constant‐temperature evaporation^[^ ^21^ ^]^
(C_8_NH_12_)_4_Bi_0.57_Sb_0.43_Br_7_·H_2_O	Cooling‐induced crystallization^[^ ^58^ ^]^
(*N*‐methylpyrrolidinium)_3_Sb_2_Cl_9−9x_Br_9x_	Cooling‐induced crystallization^[^ ^90^ ^]^
A_2_InCl_5_ (H_2_O) (A = Rb, Cs)	Hydrothermal^[^ ^77^ ^]^
Rb_7_Bi_3_Cl_16_	Hydrothermal^[^ ^26^ ^]^
Cs_3_Sn_3_F_2_Cl_7_	Hydrothermal^[^ ^91^ ^]^
Cs_2_InBr_5_·H_2_O	Cooling‐induced crystallization^[^ ^74^ ^]^
Cs_3_Bi_2_I_9_	Cooling‐induced crystallization^[^ ^92^ ^]^
Cs_3_BiBr_6_	Cooling‐induced crystallization^[^ ^93^ ^]^
Cs_3_Cu_2_I_5_	Solvent evaporation crystallization^[^ ^49^ ^]^
Cs_4_EuX_6_ (X = Br, I)	Vertical Bridgman^[^ ^35^ ^]^
Cs_4_PbBr_6_	Antisolvent vapor crystallization^[^ ^83^ ^]^
**Perovskite NCs**	
Cs_4_PbBr_6_	Reverse microemulsion method,^[^ ^29^, ^94^ ^]^ supersaturated recrystallization,^[^ ^95^ ^]^ hot injection^[^ ^38^, ^96^ ^]^
Cs_4_SnBr_6_	Supersaturated recrystallization,^[^ ^97^ ^]^ hot injection^[^ ^34^, ^44^ ^]^
Cs_4_KSnBr_6_	Supersaturated recrystallization^[^ ^97^ ^]^
Cs_3_Bi_2_I_9_	Hot injection^[^ ^39^, ^98^ ^]^
**Perovskite thin films**	
(CH_3_NH_3_)_3_Bi_2_I_9_	One‐step spin‐coating,^[^ ^20^, ^55^, ^99^ ^]^ two‐step spin‐coating,^[^ ^52^ ^]^ solvent engineering^[^ ^52^ ^]^
Cs_3_Sb_2_I_9_	One‐step spin‐coating^[^ ^100^ ^]^
Cs_3_Bi_2_I_9_	One‐step spin‐coating^[^ ^54^ ^]^
Cs_2_TeI_6_	One‐step spin‐coating^[^ ^93^ ^]^
(CH_3_NH_3_)_3_Sb_2_I_9_	Two‐step spin‐coating,^[^ ^52^ ^]^ solvent engineering^[^ ^52^ ^]^

### 3.1 Single‐Crystal Growth Methods

**Figure 6:**
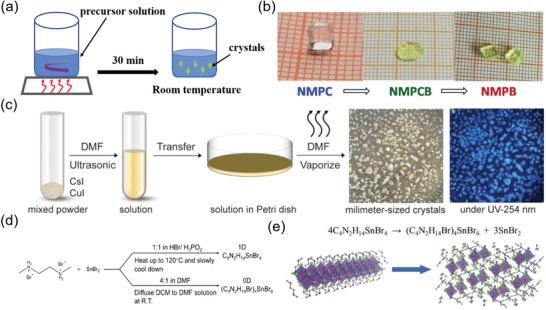
a) Schematic illustration of cooling‐induced crystallization method to grow 0D perovskite single crystals. b) Color change of block crystals of NMPC (*x* = 0), NMPCB (*x* = 1/3), and NMPB (*x* = 1). Reproduced with permission.^[^ ^90^ ^]^ Copyright 2017, American Chemical Society. c) Schematic illustration of growing Cs_3_Cu_2_I_5_ perovskite crystals via room temperature solvent evaporation crystallization method, with photographs under day light and UV lamp on the right. Reproduced with permission.^[^ ^49^ ^]^ Copyright 2020, Wiley‐VCH. d) The synthetic process of 1D and 0D tin‐based perovskites single crystals. e) Photoinduced structural transformation from 1D to 0D structure. Reproduced with permission.^[^ ^89^ ^]^ Copyright 2017, Wiley‐VCH.

Perovskite single crystals have a large carrier diffusion length >175 µm under 1 sun illumination,^[^ ^101^ ^]^ long carrier lifetime,^[^ ^102^ ^]^ low trap density (≈10^9^–10^10^ cm^−3^),^[^ ^103^ ^]^ good environment stability, and have no grain boundaries, which are beneficial to reduce carrier recombination and promote carrier extraction efficiency.^[^ ^104^ ^]^ Therefore, perovskite single crystals are not only ideal states for analyzing intrinsic optoelectronic properties, but also candidates to achieve high performance optoelectronics.^[^ ^101^ ^]^ To date, 0D perovskite single crystals have been successfully synthesized by using various methods. Cooling‐induced crystallization method is a common method to obtain single crystals through lowering the temperature to decrease the solubility of saturated precursor solution. In 1996, Kawai et al. synthesized 0D (CH_3_NH_3_)_3_Bi_2_I_9_ single crystals by mixing amounts of (BiO_2_)CO_3_ and CH_3_NH_2_ in concentrated aqueous solution of HI to form a saturated solution, then cooling this saturated solution from 80 °C to room temperature.^[^ ^40b^ ^]^ Utilizing the same synthetic method, Zhang et al. obtained 0D mixed metal halide perovskite (C_8_NH_12_)_4_Bi_0.57_Sb_0.43_Br_7_·H_2_O single crystal.^[^ ^58^ ^]^ The single crystal was formed by stirring the mixture of equal amounts of BiBr_3_, SbBr_3_, and C_8_NH_12_Br at 100 °C for 30 min, then stopping stirring and cooling the mixture to room temperature, as shown in **Figure** **6a**. Compared with the pristine perovskite (C_8_NH_12_)_4_BiBr_7_·H_2_O that has a narrow emission, the (C_8_NH_12_)_4_Bi_0.57_Sb_0.43_Br_7_·H_2_O mixed perovskite shows ultra‐broadband emission ranging from 400 to 850 nm, higher PL QY and excellent air stability in the case of retaining the 0D crystal structure. With the similar method, Ji et al. prepared a class of lead‐free 0D perovskite single crystals, (*N*‐methylpyrrolidinium)_3_Sb_2_Cl_9−9_ *_x_* Br_9_ *_x_* (*x* = 0–1) (NMPX) that grew from the saturated aqueous solutions of *N*‐methylpyrrolidinium chloride, Sb_2_O_3_, HCl, and HBr.^[^ ^90^ ^]^ The NMPX shows tunable optical bandgaps and different light‐absorbing properties, which are correspond well to the color change of these block crystals of NMPC (*x* = 0), NMPCB (*x* = 1/3), and NMPB (*x* = 1), as shown in Figure 6b. In addition, they found that partial and/or full substitution of halogen can retain the 0D structure and bulk ferroelectricity.

Recently, Zheng et al. grew MA_3_Bi_2_I_9_ perovskite single crystals via seed‐crystal‐assisted constant‐temperature evaporation method.^[^ ^21^ ^]^ First, the mixture of MAI and BiI_3_ with a molar ratio of 3:2 dissolved in *γ*‐butyrolactone (GBL) solution was heated to 60 °C for 12 h and filtered with a PTFE before use. Then the precursor solution was kept at 60 °C for 24 h to obtain the MA_3_Bi_2_I_9_ single crystal seeds, following by adding one crystal seed into fresh precursor solution at 70 °C for 20 days. Finally, a reddish black MA_3_Bi_2_I_9_ crystal with a large size of 27 mm was obtained. As‐prepared single crystal exhibits excellent thermal and ambient stability. Traditional hydrothermal method is also a widely used method for synthesizing single crystals. Gong et al. synthesized 0D Cs_3_Sn_3_F_2_Cl_7_ single crystals through this hydrothermal method.^[^ ^91^ ^]^ The mixture of CsF and SnCl_2_·H_2_O dissolved in H_2_O was sealed in an autoclave equipped with a Teflon liner and heated at 220 °C for 1 day. Then, it was slowly cooling to the room temperature at a rate of 3 °C h^−1^. Finally, the colorless transparent crystals were obtained by washing the reacting product with deionized water and ethanol. Using the same method, Xie et al. synthesized 0D Rb_7_Bi_3_Cl_16_ single crystals by heating the mixture of RbCl and BiCl_3_ dissolved in HCl at 160 °C for 24 h in a Teflon‐lined stainless steel autoclave and then slowly cooling the mixture to room temperature at a freezing rate of 2 °C h^−1^ and washing with water.^[^ ^26^ ^]^

In addition to changing the temperature of precursor solution, solvent evaporation crystallization is also a common method to grow single‐crystalline materials by precipitating the precursor from solution through evaporating solvent.^[^ ^105^ ^]^ Using this method, Zhang et al. first prepared the 0D copper based all‐inorganic lead‐free perovskites Cs_3_Cu_2_I_5_ single crystals.^[^ ^49^ ^]^ CsI and CuI were dissolved in dimethyl formamide (DMF) by ultrasonication treatment in a short time. Then the mixture was put in a petri dish and transferred to fume hood to evaporate the DMF solvent. Finally, a millimeter‐sized Cs_3_Cu_2_I_5_ crystal was obtained overnight (Figure 6c). As‐prepared Cs_3_Cu_2_I_5_ single crystal shows bright blue light emission with high PL QY up to 89% under UV lamp (254 nm). In addition, the size of crystals can be adjusted by controlling the concentration of precursors in DMF solution.

Antisolvent vapor crystallization is another effective method to grow single crystals. In this method, antisolvent is added in saturated solution system to mix well. The addition of antisolvent can reduce the solubility of solute, resulting in the solute precipitates. Ma's group prepared 0D (C_4_N_2_H_14_Br)_4_SnBr_6_ perovskite single crystals using this method. As shown in Figure 6d, C_4_H_14_N_2_Br_2_ and SnBr_2_ were dissolved in DMF with the ratio of 4:1 to form a clear precursor solution. Then diffusing dichloromethane antisolvent into the prepared DMF solution at room temperature and keeping them overnight, then the (C_4_N_2_H_14_Br)_4_SnBr_6_ perovskite single crystals were obtained.^[^ ^89^ ^]^ As‐prepared 0D (C_4_N_2_H_14_Br)_4_SnBr_6_ single crystal shows bright yellow emission under UV irradiation. In addition, they also prepared 1D C_4_N_2_H_14_SnBr_4_ perovskite crystals without light emission by stirring the solution of equivalent SnBr_2_ and C_4_H_14_N_2_Br_2_ in the mixture of HBr and H_3_PO_2_ with the ratio of 1:1 at 120 °C, and lowing this solution to room temperature when bromide salts was completely dissolved (Figure 6e). Interestingly, the 1D crystal structure can partially transform into 0D structure upon continuous UV irradiation, as shown in Figure 6e, which is ascribed to photon‐induced photodissociation of metal halide bonds. They also prepared 0D mixed‐halide (C_4_N_2_H_14_Br)_4_SnBr_3_I_3_ perovskite single crystals by the similar method.^[^ ^69^ ^]^ This mixed‐halide perovskite single crystal exhibits better thermal stability and photostability than the pure‐halide counterparts, which may be caused by the enhanced nonradiative decay and reduced ion diffusion in the mixed‐halide perovskite single crystal due to its 0D structure.

The vertical Bridgman method was reported by Wu et al. to prepare Cs_4_EuX_6_ (X = Br, I) single crystals.^[^ ^35^ ^]^ The mixtures of CsX and EuX_2_ (X = Br, I) were loaded into quartz ampoules, and they were evacuated to 10^−6^ mbar and heated to 250 °C for 10 h to remove residual water and oxygen impurities. Then the sealed ampoules were transferred to the Bridgman growth furnace to grow crystals by cooling the furnace to room temperature. As‐prepared Cs_4_EuI_6_ single crystal has a cloudy layer on the crystal surface, which is caused by the deposition of volatile precursor materials during cooling process. However, this phenomenon is not observed in Cs_4_EuBr_6_ single crystal. The thin slabs cut from the Cs_4_EuBr_6_ and Cs_4_EuI_6_ crystals are transparent and crack‐free. These 0D Cs_4_EuX_6_ (X = Br, I) perovskite crystals are self‐activated blue emitters with slight hygroscopicity and high radiation detection efficiency.

### 3.2 Colloidal Synthesis

**Figure 7:**
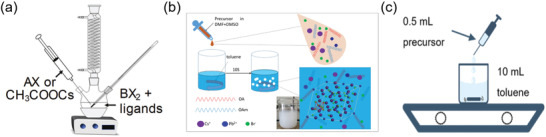
a) Synthesis of perovskite NCs by the hot‐injection method. Reproduced with permission.^[^ ^108^ ^]^ Copyright 2020, The Royal Society of Chemistry. b) Schematic illustration of synthesis process of pure 0D Cs_4_PbBr_6_ NCs at room temperature, with the products images suspended in toluene solution shown in the insets. Reproduced with permission.^[^ ^95^ ^]^ Copyright 2017, Springer Science. c) Schematic illustration of room temperature antisolvent method. Reproduced with permission.^[^ ^97^ ^]^ Copyright 2020, American Chemical Society.

Colloidal metal halide perovskite NCs have emerged as promising materials for optoelectronic devices due to their high PL QY reaching about 100%,^[^ ^106^ ^]^ and adjustable optoelectronic properties by the composition, size, and dimensionality optimization.^[^ ^107^ ^]^ The perovskite NCs can be prepared through facile synthesis procedures mainly including hot‐injection and room‐temperature synthesis. Hot‐injection method is carried out to produce colloidal perovskite NCs with high quality by injecting Cs‐oleate precursor into PbX_2_ precursor at high temperature, following by fast cooling in an ice‐water bath, as shown in **Figure** **7a**. Pal et al.^[^ ^39^ ^]^ used hot injection method to synthesize the 0D Cs_3_Bi_2_I_9_ NCs. First, Cs‐oleate precursor was prepared by dissolving Cs_2_CO_3_ in octadecene and oleic acid (OA) at 150 °C. Then this Cs‐oleate precursor was quickly injected into the mixture of BiI_3_, octadecene, OA and OLA at 180 °C under N_2_ atmosphere. Ten seconds later, the reaction mixture was cooled down to room temperature by an ice‐water bath, and finally a bright red solution of Cs_3_Bi_2_I_9_ NCs was obtained. Later, Tan et al.^[^ ^34^ ^]^ synthesized 0D Cs_4_SnX_6_ (X = Br, I) NCs by swiftly injecting SnX_2_ precursor dissolved in tri‐*n*‐octylphosphine into the prepared Cs‐precursor at 210 °C for Cs_4_SnBr_6_ NCs and 250 °C for Cs_4_SnI_6_ NCs, following by cooling these reactants down to room temperature by an ice‐water bath. For the hot‐injection approach, there are various choices of precursor and more homogeneous diffusion of reactants, but the temperature is an essential factor to affect the growth of NCs.

Compared with high‐temperature hot injection, room‐temperature synthesis is more simple and low cost due to the low synthesis temperature and undemanding synthesis environment. Reverse microemulsion method was first applied to the synthesis of 0D Cs_4_PbBr_6_ NCs by Zhang et al.^[^ ^29^ ^]^ First, Cs‐oleate precursor was prepared by stirring the mixture of Cs_2_CO_3_ and OA at 130 °C under vacuum for 1 h. Then, Cs‐oleate precursor, n‐hexane, and OA were mixed in a three‐neck flask under the mild degassing and nitrogen purging. Finally, a mixture of PbBr_2_ dissolved in DMF, HBr, OA, and OLA was swiftly injected into the flask, followed by the color changing from pale‐white to green in 10 min, indicating the formation of Cs_4_PbBr_6_ NCs. As‐synthesized Cs_4_PbBr_6_ NCs exhibit high PL QY of 65% in colloidal state and 54% in the form of thin films. Later, Yang et al. synthesized Cs_4_PbBr_6_ NCs with varied sizes via supersaturated recrystallization method at room temperature (Figure 7b).^[^ ^95^ ^]^ 0.8 mmol of CsBr and 0.4 mmol of PbBr_2_ were dissolved in the mixture of 5 mL DMF and 5 mL dimethyl sulfoxide (DMSO), followed by adding OA and OLA to stabilize the precursor solution. Then, 10 mL precursor solutions were quickly injected into 100 mL toluene under vigorous stirring, and the reaction was finished after 10 s. The combined use of DMF and DMSO increased the Cs^+^ precursor concentration, and the dissolving capacity of toluene for those ions (Cs^+^, Pb^2+^, Br^−^) is lower than the mixture of DMF and DMSO, leading to the fast crystallization of Cs_4_PbBr_6_ NCs. The size of NCs can be adjusted from 150 to 350 nm through simply regulating the amounts of ligands. While no excitonic emission was observed from the prepared Cs_4_PbBr_6_ NCs in this work. Recently, Zhang et al.^[^ ^97^ ^]^ synthesized 0D Cs_4_SnBr_6_ and Cs_3_KSnBr_6_ perovskites via similar supersaturated recrystallization method at room temperature by dropping the precursor solution dissolved in DMF into toluene solution under quickly stirring (Figure 7c).

### 3.3 Thin Films

**Figure 8:**
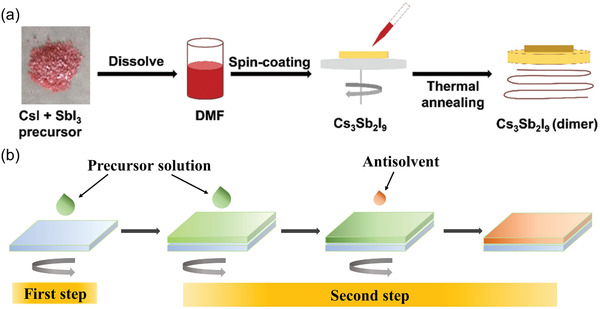
a) Schematic illustration of the one‐step spin‐coating method to prepare 0D Cs_3_Sb_2_I_9_ film. Reproduced with permission.^[^ ^100^ ^]^ Copyright 2019, Wiley‐VCH. b) Schematic illustration of two‐step spin‐coating method to prepare 0D perovskite film.

High‐quality thin films are important to achieve high‐performance devices. The most common method to obtain perovskite thin films is to spin‐coating precursor solutions on different matrices, following by annealing treatment. Öz et al. reported 0D (CH_3_NH_3_)_3_Bi_2_I_9_ thin film by dissolving CH_3_NH_3_I and BiI_3_ at a 3:2 molar ratio in anhydrous DMF to form a clear red solution, among which the CH_3_NH_3_I was prepared by reacting methylamine with hydroiodic acid in water at 0 °C for 2 h. Then the solution was heated to 60 °C and spin coated on the substrate to form 0D (CH_3_NH_3_)_3_Bi_2_I_9_ thin film.^[^ ^55^ ^]^ With a similar method, Umar et al. prepared 0D Cs_3_Sb_2_I_9_ perovskite film.^[^ ^100^ ^]^ SbI_3_ and CsI were dissolved in DMF at a molar ratio of 2:3 as precursor. The precursor was then spun onto TiO_2_/FTO substrates and followed by an annealing treatment, thus a yellow Cs_3_Sb_2_I_9_ film was obtained (**Figure** **8a**). This one‐step spin‐coating method is very simple and relatively common for most perovskite film preparation, but various chemical reactions may occur in the solution to influence the quality of resulting film. Alternatively, the two‐step spin‐coating method can improve the film quality. Hebig et al. fabricated (CH_3_NH_3_)_3_Sb_2_I_9_ perovskite thin film by two‐step spin‐coating method. First, SbI_3_ and MAI with a molar ratio of 2:3 were added in a mixture of GBL:DMSO with an overall concentration of 35 wt% to form the precursor solution.^[^ ^52^ ^]^ Then the precursor solution was spin‐coated on quartz glass substrates at 3000 rpm for 10 s in the first step. In the second step, the precursor solution was spin coated on top of the layer obtained in the first step at 5000 rpm for 20 s. In addition, they dropped toluene as antisolvent on the top of the sample in 10 s before the end of second step, as shown in Figure 8b. Consequently, the resulting thin films show a very flat and homogeneous surface.

## 4 Applications of 0D Perovskites

0D perovskites have unique isolated structure and thus show intriguing properties, such as large exciton binding energy, broadband emissions and good ambient stability. These properties make 0D perovskites have potential in the fabrication of optoelectronic devices, including LEDs, solar cells, photodetectors, X‐ray detectors, scintillators, luminescent solar concentrators (LSCs), electrochemical capacitors, and lasers.

### 4.1 LEDs

At presently, 3D lead halide perovskites have been widely used in light‐emitting applications due to their outstanding intrinsic features, including high PL QY ≈ 100%, bipolar carrier transport, narrow FWHM, and wide color gamut.^[^ ^109^ ^]^ However, the toxicity of lead and unsatisfactory stability limited their practical applications.^[^ ^110^ ^]^ By contrast, 0D perovskites show excellent stability due to their individual metal halide octahedra. In addition, the broadband emission and low self‐absorption makes them have great potential in down conversion white LED applications as phosphors.

**Figure 9:**
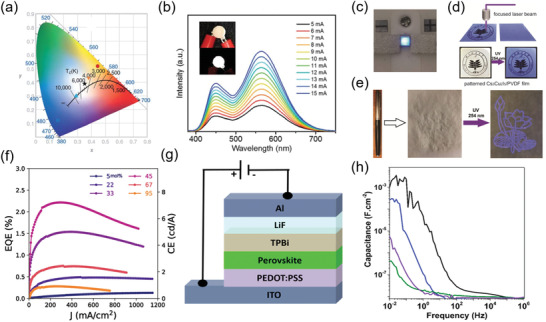
a) CIE color coordinates and CCTs for 0D (C_4_N_2_H_14_Br)_4_SnBr_6_‐based white LEDs. b) Emission spectra of the white LED at different driving currents, with a photo of this device in off and on state in the insets. a,b) Reproduced with permission.^[^ ^18^ ^]^ Copyright 2018, The Royal Society of Chemistry. c) Photograph of the UV‐pumped blue LED based on the Cs_3_Cu_2_I_5_ phosphor. d) Schematic illustration of direct laser writing technology (top), and photographs of a patterned Cs_3_Cu_2_I_5_/PVDF film under daylight and UV lamp (bottom). e) Photographs of a weighing paper drew by Cs_3_Cu_2_I_5_ ink under daylight and UV lamp. c‐e) Reproduced with permission.^[^ ^49^ ^]^ Copyright 2020, Wiley‐VCH. f) PLQY, conductivity of the composite films and EQE of corresponding devices. Reproduced with permission.^[^ ^111^ ^]^ Copyright 2019, Wiley‐VCH. g) Device structure of KBr‐mixed 0D/3D perovskite LEDs. h) Capacitance–frequency plot of impedance spectroscopy carried out on each LED devices with 0D/3D: KBr v/v ratio of 100:0 (black line), 90:10 (blue line), 80:20 (green line), and 70:30 (purple line), respectively. g,h) Reproduced with permission.^[^ ^112^ ^]^ Copyright 2020, American Chemical Society.

Zhou et al. used 0D (C_4_N_2_H_14_Br)_4_SnBr_6_ with highly efficient yellow emission as phosphors, blending with blue emitting europium‐doped barium magnesium aluminates (BaMgAl_10_O_7_:Eu^2+^) to fabricate downconversion white LEDs, in which a UV LED chip (340 nm) was used to excite the yellow and blue phosphors.^[^ ^18^ ^]^ By controlling the blending ratio of these two phosphors, white emission LEDs with a cover range from “cold” to “warm” were obtained (**Figure** **9a**), and this white LED shows great stability at different operating currents (Figure 9b). To avoid the oxidation of Sn^2+^ into Sn^4+^ and further improve the stability of perovskites, Tan et al. used Sn^4+^ to replace Sn^2+^ and synthesized blue emitting Bi^3+^ doped Cs_2_SnCl_6_ 0D perovskite.^[^ ^50^ ^]^ Then a white LED was fabricated by coating the mixture of blue emitting Cs_2_SnCl_6_: Bi perovskites and yellow phosphors (Ba_2_Sr_2_SiO_4_:Eu^2+^ and GaAlSiN_3_:Eu^2+^) on a 365 nm LED chip. The obtained LED emitted bright and warm white light with color temperature (CCT) of 4486 K and Commission Internationale de I'Eclairage (CIE) color coordinate of (0.36, 0.37). Zhang et al. fabricated UV pumped blue phosphor LEDs (Figure 9c) with a CIE coordinate (0.154, 0.118) by using the Cs_3_Cu_2_I_5_ powder as solid‐state phosphor. In addition, they used the solution of Cs_3_Cu_2_I_5_ dissolved in DMF as ink to write hidden characters that are visible under UV light (Figure 9e), which can be used for anticounterfeit and encryption applications.^[^ ^49^ ^]^ Furthermore, they used direct laser writing technology to fabricate a patterned film based on Cs_3_Cu_2_I_5_/PVDF composites (Figure 9d), suggesting their potential for display application.

The reported 0D perovskite LED are almost photoluminescent LED, and there are few reports about electroluminescent LED based on pure 0D perovskites. Jun et al. reported a blue LED based on 0D Cs_3_Cu_2_I_5_ with the structure of ITO/ZSO/Cs_3_Cu_2_I_5_/NPD/MoO*_x_*/Ag.^[^ ^41^ ^]^ The peak wavelength of electroluminescence is 440 nm, which is almost the same as that of PL. However, the maximum luminance of the LED was very low (about 10 cd m^−2^). The possible obstacle is the large bandgap of 0D perovskites caused by their localized electronic structure, which would lead to low carrier mobility and poor charge injection in perovskite LED. The 0D/3D admixture perovskites were investigated to exhibit improved performance in electroluminescent LED than 3D perovskite based one. Lian et al. fabricated perovskite LEDs by using CsPbBr_3_/Cs_4_PbBr_6_ composites as the emissive layer.^[^ ^111^ ^]^ By tuning the ratio and thickness of the CsPbBr_3_/Cs_4_PbBr_6_ composite perovskite layer, the LEDs achieved 55 000 cd m^−2^ in the maximum brightness and 2.5% in EQE, which is tenfold enhancement compared with pure CsPbBr_3_ based LEDs (Figure 9f). The enhanced performance is attributed to the passivated surfaces of CsPbBr_3_ by Cs_4_PbBr_6_ and the enhanced carrier radiative recombination process due to the spatial confinement of CsPbBr_3_ grains within Cs_4_PbBr_6_ host lattice. In addition, this CsPbBr_3_/Cs_4_PbBr_6_ composites‐based LED shows significant improvement in stability, which can be attributed to the suppressed ion migration, resulting from the isolated structure of 0D perovskite. Afterward, Kanwat et al. added KBr in CsPbBr_3_/Cs_4_PbBr_6_ composite perovskite. Thus, the KBr‐mixed 0D/3D perovskite composed of 0D Cs_4−_ *_x_* K*_x_* PbBr_6_ and 3D CsPbBr_3_ with a ratio of 0.35:0.65 was formed, together with some unreacted KBr passivating the surface and grain boundaries of the 3D perovskite.^[^ ^112^ ^]^ Then they used KBr‐mixed 0D/3D perovskite as emissive layer to fabricate the electroluminescent LEDs with a device architecture of ITO/PEDOT:PSS/perovskite/TPBi/LiF/Al, as shown in Figure 9g. Compared with the unmodified device, the KBr‐modified perovskite devices achieved 14‐fold increase in the device efficiencies, resulting from the passivation of the resultant KBr. Additionally, the introduction of KBr suppressed the ion migration and accumulation at the interfaces, resulting in 30‐fold improvement in the stability of the KBr‐modified perovskite LED compared with the unmodified device. Furthermore, they measured the capacitance of perovskite LEDs with different KBr concentration and observed a remarkable reduction in the capacitance with KBr concentration increasing (Figure 8h), which further illustrates K^+^‐doping could suppress ion migration and accumulation at the interfaces between perovskite emissive layer and HTL. Recently, Bao et al. synthesized high‐quality CsPbBr_3_@Cs_4_PbBr_6_ NCs by adding SnBr_2_ in the synthesis process of CsPbBr_3_. The extra Br^−^ ions provided by SnBr_2_ and the reduced Pb^2+^ ions promoted the formation of Cs_4_PbBr_6_.^[^ ^113^ ^]^ Benefitting from the passivation of Cs_4_PbBr_6_ to CsPbBr_3_, the high‐efficiency perovskite LEDs were achieved by using the as‐synthesized CsPbBr_3_@Cs_4_PbBr_6_ NCs as emitting layer. The highest current efficiency and EQE of this LED reached 4.89 cd A^−1^ and 1.74%, respectively, which is the best performance of CsPbBr_3_@Cs_4_PbBr_6_ perovskite‐based LED device. In addition to the 0D/3D admixture perovskites, the 0D/1D admixture has also been investigated for electroluminescent LED. Chen et al. reported efficient and bright warm‐white electroluminescent LED based on a mixture of 0D Cs_3_Cu_2_I_5_ and 1D CsCu_2_I_3_ enabled by introducing an organic additive (Tween, polyethylene glycol sorbitan monooleate) into the precursor solutions.^[^ ^114^ ^]^ The chemical interaction between the C—O—C bond in Tween and Cs reduced the trap states, improving the PL QY and the surface potential of the films, and facilitating the charge transport in the LEDs. Consequently, this LED achieved an EQE of 3.1% and a luminance of 1570 cd m^−2^ at a low voltage of 5.4 V. Moreover, this LED had a CIE color coordinate of (0.44, 0.53) and a CCT of 3650 K in the warm‐white region.

### 4.2 Solar Cells

Up to date, perovskite solar cells based on 3D perovskites have undergone rapid progress reaching 25.5% efficiencies.^[^ ^4^ ^]^ However, these 3D perovskites‐based solar cells suffer from the instability in ambient environment, the toxicity from lead, and rate‐dependent current–voltage hysteresis. The hysteresis observed in 3D perovskites is mainly attributed to ion migration and charge trapping at interfaces and grain boundaries,^[^ ^115^ ^]^ which has brought many difficulties for the accurate measurement of device performances and seriously affected the stability of perovskite solar cells.^[^ ^116^ ^]^ Compared to 3D perovskites, 0D perovskites possess isolated metal halide octahedrons, which could inhibit ion migration between metal halide octahedrons, and thus leading to low hysteresis.

**Figure 10:**
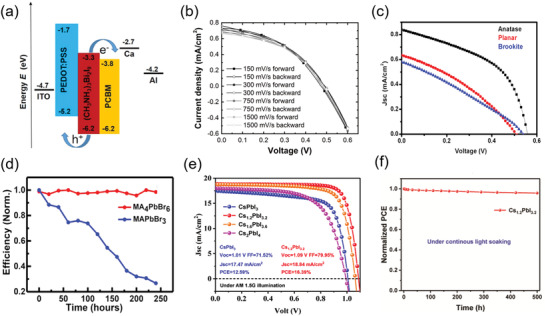
a) Energy level diagram of the 0D (CH_3_NH_3_)_3_Bi_2_I_9_ perovskite‐based solar cells. Reproduced with permission.^[^ ^55^ ^]^ Copyright 2016, Elsevier. b) *J*–*V* characteristic measured in the forward and backward scan direction for varying scan rates between 150 mV s^−1^ and 1500 mV s^−1^. Reproduced with permission.^[^ ^20^ ^]^ Copyright 2017, The Royal Society of Chemistry. c) *J*–*V* curve of (CH_3_NH_3_)_3_Bi_2_I_9_‐based devices in both planar and mesoporous device architectures. Reproduced with permission.^[^ ^99^ ^]^ Copyright 2016, American Chemical Society. d) The stability comparation between the devices based on 0D MA_4_PbBr_6_ and 3D MAPbBr_3_ under constant illumination and 65% humidity. Reproduced with permission.^[^ ^117^ ^]^ Copyright 2019, American Chemical Society. e) *J*–*V* curves of the devices using Cs_1+_ *_x_* PbI_3+_ *_x_* as the absorber layer. f) Light‐soaking stability measurement of the device based on Cs_1.2_PbI_3.2_. e,f) Reproduced with permission.^[^ ^118^ ^]^ Copyright 2019, Wiley‐VCH.

Oez et al. first used 0D perovskite (CH_3_NH_3_)_3_Bi_2_I_9_ as absorber layer to fabricate a solar cell with a planar heterojunction configuration (ITO/PEDOT/(CH_3_NH_3_)_3_Bi_2_I_9_/PCBM/Ca/Al) (**Figure** **10a**).^[^ ^55^ ^]^ Due to the large exciton binding energy (400 mV), wide bandgap (2.9 eV) of the (CH_3_NH_3_)_3_Bi_2_I_9_ perovskites, and mismatched energy level with other functional layers, the solar cell exhibits low PCE of being close to 0.1%. Then, they proposed that further study on the reduction of bandgaps and exciton binding energy of (CH_3_NH_3_)_3_Bi_2_I_9_, which will further improve the performance of 0D perovskite solar cells. Calum et al. fabricated 0D (CH_3_NH_3_)_3_Bi_2_I_9_ perovskite solar cells with the structure ITO/compact‐TiO_2_/mesoporous‐TiO_2_/(CH_3_NH_3_)_3_(Bi_2_I_9_)/Spiro‐MeOTAD/Au or Ag.^[^ ^20^ ^]^ Very low hysteresis in the broad scanning rate range of 150 to 1500 mV s^−1^ was observed in this solar cell (Figure 10b), because the 0D structure of spatially separated Bi_2_I_9_ bioctahedra inhibited I^−^ ion migration among Bi_2_I_9_ bioctahedra. This is an encouraging result for a mesoscopic solar cell without any interfacial layer engineering. Later, Singh et al. reported (CH_3_NH_3_)_3_Bi_2_I_9_ perovskite solar cells with planar and mesoporous (anatase and brookite TiO_2_) device structures.^[^ ^99^ ^]^ They found that the solar cell based on anatase TiO_2_ mesoporous layer shows better photovoltaic performance than planar and brookite mesoporous layer based devices, as shown in Figure 10c, resulting from the more uniform morphology of (CH_3_NH_3_)_3_Bi_2_I_9_ on anatase mesoporous layer due to the rich pores on it. Therefore, as‐fabricated devices show improved performance with 0.2% PCE compared with that of 0.1% for planar layer‐based devices and 0.09% for brookite layer‐based devices, and good stability for 10 weeks in ambient condition. Sharma et al. fabricated photovoltaic devices based on 0D MA_4_PbBr_6_ perovskite absorber layer, in which mesoporous TiO_2_ and spiro‐OMeTAD are utilized as the electron transport layer and hole transport layer, respectively.^[^ ^117^ ^]^ They demonstrated that water can induce MA_4_PbBr_6_ to transform into MAPbBr_3_ under MABr rich conditions, since the reaction between water and methyl ammonium ion leads to the coupling and reorganization of the lattice of 0D MA_4_PbBr_6_ to form the 3D MAPbBr_3_ perovskites. And this transformed 3D perovskite displays excellent photo‐ and thermal stability, due to the protection of the excess MABr around the MAPbBr_3_ grains. Therefore, under the same conditions with 65% humidity, the devices based on 0D MA_4_PbBr_6_ exhibit higher efficiencies and better stability than that of direct 3D MAPbBr_3_ perovskites‐based devices (Figure 10d),

Though 0D perovskite solar cells exhibit enhanced stability, the obtained devices suffer from low device efficiency. Recently, Bai et al. designed 0D/3D heterostructure perovskites consisting of Cs_4_PbI_6_ and CsPbI_3_ by tuning the stoichiometry of the precursors between CsI and PbI in a typical one‐step spin‐coating method.^[^ ^118^ ^]^ The 0D Cs_4_PbI_6_ formed in suit as a wrapping layer is distributed around the 3D CsPbI_3_ to simultaneously passivate the defects and keep the phase stability of CsPbI_3_. Furthermore, the solar cell based on the optimized perovskite films exhibits improved device performance and good stability, as shown in Figure 10e,f. Therefore, 0D perovskites can serve as a protect layer to improve the device performance of perovskite solar cells.

### 4.3 Detectors

**Figure 11:**
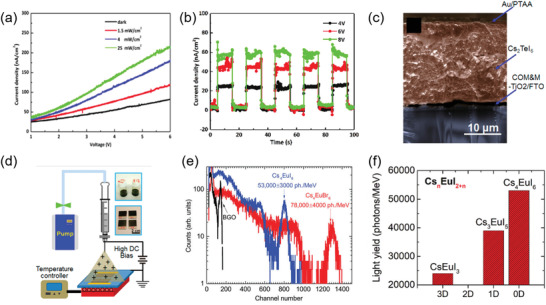
a) *I*–*V* characteristics of 0D Cs_3_BiBr_6_‐based photodetector under the dark and light illumination with different light densities. b) Photocurrent responses under various bias voltages with fixed light density of 25 mW cm^−2^. a,b) Reproduced with permission.^[^ ^122^ ^]^ Copyright 2019, The Royal Society of Chemistry. c) Cross‐sectional SEM image of X‐ray detector with a device structure of glass/FTO/c‐&m‐TiO_2_+Cs_2_TeI_6_/PTAA/Au. d) Schematic of the electrostatic‐assisted spray deposition process, with the corresponding Cs_2_TeI_6_ solutions and films shown aside. c,d) Reproduced with permission.^[^ ^93^ ^]^ Copyright 2018, American Chemical Society. e) ^137^Cs pulse height spectra of Cs_4_EuBr_6_ and Cs_4_EuI_6_ single crystals coupled to a Hamamatsu R2059 PMT. f) The variation of scintillation light yield of Cs*_n_* EuI_2+_ *_n_* (*n* = 1, 3, and 4) with different perovskite dimension. e,f) Reproduced with permission.^[^ ^35^ ^]^ Copyright 2018, The Royal Society of Chemistry.

Photodetector is a kind of device that can convert optical signals to electrical signals via the photoelectric effect, which has wide applications in many fields, including imaging, machine vision, and digital display technology.^[^ ^119^ ^]^ In recent years, perovskite materials have been used as active layers in photodetectors and exhibited excellent photodetection performances.^[^ ^120^ ^]^ However, 3D perovskite photodetectors suffer from current–voltage hysteresis, unreliable performance, and instability.^[^ ^121^ ^]^ 0D perovskites with excellent stability are promising materials for photodetectors to overcome the aforementioned limitations. Tang et al. reported a photodetector fabricated with 0D nontoxic single crystalline perovskite Cs_3_BiBr_6_ deposited on ITO electrodes.^[^ ^122^ ^]^ This photodetector shows increased photocurrent with the increase of the light density and voltage (**Figure** **11a**), and the device can repeatedly produce stable photocurrent signals in response to periodical light ON and OFF (Figure 11b). Furthermore, this photodetector achieved ultralow dark current of 0.3 nA under 6 V bias voltage and high detectivity of 0.8 × 10^9^ Jones under ambient conditions, demonstrating the potential of this material in photodetector application. In addition, the 0D Cs_3_BiBr_6_ displays high stability against temperature and moisture, which helps to improve the stability of photodetector.

0D perovskites are also potential materials for X‐ray detectors that can convert X‐ray photons into electrical signal. X‐ray detectors have been widely applied in medical radiography and security screening. To reduce health risks, the X‐ray detectors need a high sensitivity to detect the weak X‐ray dose rates under shorter exposure times. Considering the above requirements, 3D perovskites are promising candidate for X‐ray detectors, but their ion migration and high carrier concentration would cause dark current drift and unstable detector operation.^[^ ^123^ ^]^ While the isolated structure of 0D perovskites can effectively disrupt the ion migration channels, and thus suppressed ion migration. In addition, the more localized electronic structures of 0D perovskites can decrease the dark carrier concentration in comparation to 3D perovskites, leading to stable dark current and long‐term operation stability for X‐ray detectors.^[^ ^124^ ^]^ Xu et al. fabricated a planar‐configuration X‐ray detector based on 0D Cs_2_TeI_6_ perovskites thick film.^[^ ^93^ ^]^ A multilayer structure with compact and mesoporous TiO_2_ layers spin‐coated on FTO was designed to prevent shorting paths of direct contact between the top and bottom electrodes. The cross‐sectional SEM image of this device is shown in Figure 11c. The Cs_2_TeI_6_ perovskites thick film was deposited via a low‐temperature process using electrostatic‐assisted spray (E‐spray) technique under atmospheric conditions, as shown in Figure 11d. The morphology and thickness of the Cs_2_TeI_6_ films can be tuned by adjusting the temperature and E‐spray parameters. As‐prepared Cs_2_TeI_6_ perovskites thick films have a high resistivity of 4.2 × 10^10^ Ω cm due to the electronic structure and high formation energy of intrinsic iodine vacancies, which reduces shallow‐level defects that can provide parasitic free charge carriers.^[^ ^125^ ^]^ Therefore, the X‐ray detector based on 0D Cs_2_TeI_6_ thick film shows high air and moisture stability, and high sensitivity of 19.2 µC Gy_air_^−1^ cm^−2^, which is ≈20 times higher than that of the hybrid 3D perovskite polycrystalline film‐based X‐ray detectors. Recently, Zhang et al. reported an ultrasensitive and stable X‐ray detector based on 0D MA_3_Bi_2_I_9_ single crystals with a device structure of Au/MA_3_Bi_2_I_9_/Au.^[^ ^21^ ^]^ This X‐ray detectors achieved ultrahigh sensitivity of 10620 µC Gy_air_^−1^ cm^−2^ and low limit of detection of 0.62 nGy_air_ s^−1^ due to the suppressed ion migration and reduced dark carrier concentration in 0D crystal structure of MA_3_Bi_2_I_9_, thus leading to less health risks and more frequent diagnostic imaging assays. In addition, this X‐ray detector exhibits good stability under high bias and high X‐ray dose more than 23 858.5 mGy_air_ that is nearly 200 000 times of dose of a single commercial X‐ray chest radiograph, suggesting its great value in practical application.

Besides, scintillator is an emerging application direction for metal halide perovskites, which is a kind of material that can emit photons after absorbing high energy particles or rays and plays an important role in radiation detection field.^[^ ^126^ ^]^ Compared to the traditional scintillator that can only be prepared at high temperature and is difficult to adjust luminescence in the visible spectrum,^[^ ^127^ ^]^ metal halide perovskites are facile to prepare and have tunable emission wavelength.^[^ ^128^ ^]^ Especially, 0D metal halide perovskites are promising scintillator materials due to their large Stokes shifts for efficient light out‐coupling and high ray absorptivity by heavy elements (such as Pb, Cu).^[^ ^129^ ^]^ Wu et al. reported self‐activated scintillators based on 0D Cs_4_EuX_6_ (X = Br, I) single crystals, with high radiation detection efficiency, efficient and homogeneous blue luminescence for gamma‐ray spectroscopy.^[^ ^35^ ^]^ The Cs_4_EuBr_6_ and Cs_4_EuI_6_ single crystals exhibit high light yields of 78 000 ± 4000 and 53 000 ± 3000 photons MeV^−1^, which were obtained via ^137^Cs pulse height spectra (Figure 11e) of small‐size Cs_4_EuX_6_ (X = Br, I) samples with a calibrated Hamamatsu R2059 PMT, and the light yield of Cs_4_EuBr_6_ is the highest among self‐activated scintillators reported at that time. In addition, by comparing the light yield of 3D CsEuI_3_, 1D Cs_3_EuI_5_, and 0D Cs_4_EuI_6_, they found that the decrease of perovskite dimension would lead to significant increasement in the scintillation yield (Figure 11f), indicating that the 0D perovskites with strongly localized excitons are more conducive to achieving high scintillation efficiency.

### 4.4 Other Applications

**Figure 12:**
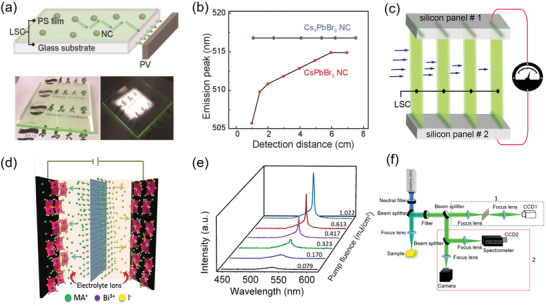
a) Schematic illustration of the LSC based on thin‐film architecture(top), and photographs of the LSC under ambient (bottom left) and one sun (100 mW cm^−2^) illumination (bottom right). b) Emission peak positions as a function of detection distance. The excitation wavelength is 400 nm. a,b) Reproduced with permission.^[^ ^94^ ^]^ Copyright 2019, Wiley‐VCH. c) Schematic of LSC prototype device composing of two silicon panels and four pieces of LSCs. Reproduced with permission.^[^ ^134^ ^]^ Copyright 2021, Elsevier. d) Schematic diagram of an electrochemical double layer capacitor based on 0D (CH_3_NH_3_)_3_Bi_2_I_9_ perovskites. Reproduced with permission.^[^ ^53^ ^]^ Copyright 2017, American Chemical Society. e) Evolution of the PL spectra of Cs_4_PbBr_6_ microcrystal with the pump fluence ranging from 0.079 to 1.022 mJ cm^−2^. f) Schematic of the optical setup for speckle‐free imaging. e,f) Reproduced with permission.^[^ ^139^ ^]^ Copyright 2019, American Chemical Society.

To meet the growing demand for renewable energy, LSCs have emerged as a large‐area sunlight collector to achieve efficient conversion from solar to electricity. A typical LSC consists of an optical waveguide (such as polymer or glass) embedded with a high‐emissive fluorophore. After absorbing sunlight, the fluorophore will re‐emit photons, which are then guided by total internal reflection toward photovoltaic devices positioned at their edges, where they are converted into electricity by photovoltaic devices.^[^ ^130^ ^]^ All‐inorganic 3D perovskite NCs are potential candidates as fluorophores in LSCs due to their excellent optical properties and high PL QY,^[^ ^131^ ^]^ but they suffer from large overlap between absorption and emission spectra, which is an obstacle to achieve high‐efficiency LSCs.^[^ ^132^ ^]^ While 0D perovskites have small absorption/emission spectral overlap (large Stokes shift) and long‐term stability, which meet the requirements of high‐efficiency LSCs. Zhao et al. reported semitransparent large‐area LSCs using 0D Cs_4_PbBr_6_ perovskite NCs as fluorophores, as shown in **Figure** **12a**.^[^ ^94^ ^]^ Compared with 3D CsPbBr_3_‐based LSCs, the 0D Cs_4_PbBr_6_‐based LSCs exhibit higher external optical efficiency of 2.4% under one sun illumination (100 mW cm^−2^) and a PCE of 1.8% under natural sun illumination (30 mW cm^−2^). The distance‐dependent PL of the LSC was then measured to demonstrate the reabsorption capacity in Cs_4_PbBr_6_ NCs and CsPbBr_3_ NCs.^[^ ^133^ ^]^ As shown in Figure 12b, the Cs_4_PbBr_6_ NCs exhibit slight spectral variation as the optical paths increase in comparison to the CsPbBr_3_ NCs with significant PL red‐shift, indicating the less reabsorption in Cs_4_PbBr_6_ NCs. In addition, the LSCs based on 0D perovskite NCs show excellent stability. This result further illustrated that 0D perovskite NCs are better candidates as emitters for LSC applications than 3D perovskite NCs. Recently, Liu et al. used Cs_4_PbBr_6_ NCs as embedding phosphor to fabricated an LSC with an edge coupling efficiency of 81% and PCE of 1.1%.^[^ ^134^ ^]^ The large Stokes shift of Cs_4_PbBr_6_ NCs up to 1.28 eV prevents them from reabsorption, which is beneficial for the LSCs. In addition, they also designed an LSC prototype device composing of two silicon panels and four LSC slides (Figure 12c). Despite the PCE (0.2%) of this device is relatively lower than that of individual LSC, this work is important for realizing real‐device coupling to LSC.

At present, developing novel, environmentally friendly, and sustainable energy storage devices have attracted much research attention due to the deteriorating environment caused by fossil fuels. Electrochemical capacitors are regarded as excellent energy storage devices owing to their long lifetime, high power density, and fast charge–discharge characteristics, which even have the potential to replace batteries in some applications.^[^ ^135^ ^]^ However, low energy density of electrochemical capacitors is an obstacle for their wide applications. Pious et al. reported an electrochemical double layer capacitor based on 0D (CH_3_NH_3_)_3_Bi_2_I_9_ perovskites.^[^ ^53^ ^]^ Compared with 3D MAPbI_3_ perovskite‐based electrochemical capacitor, the 0D (CH_3_NH_3_)_3_Bi_2_I_9_ based capacitors show higher energy density and smaller charge transport resistance due to the vacant spaces supplied by isolated [Bi_2_I_9_]^3−^ bioctahedra and large surface area of the 0D structures, which caused the easy access of electrolyte to the active material, as shown in Figure 12d. This capacitor has a capacitance of 5.5 mF cm^−2^, which is three orders of magnitude higher than that of 5.89 µF cm^−2^ of 3D MAPbI_3_ based one reported by Zhao et al.^[^ ^136^ ^]^ In addition, the capacitor retained 84.8% capacitance of its initial value even after 10 000 cycles, showing a long‐term cycling stability.

Laser is a strong coherent light source composed of a gain media, a pump source, and an optical cavity, which has been commonly used in scientific research, communication, and medicine.^[^ ^137^ ^]^ Recently, metal halide perovskites are demonstrated as excellent gain media for laser applications due to their high optical gain coefficients, widely tailorable bandgaps, high PL QY, and high charge mobility.^[^ ^138^ ^]^ However, the current research is mainly focus on 3D and 2D perovskites and the development of perovskite lasers is still in its infancy. More recently, 0D perovskites have been demonstrated to achieve lasing emission upon optical pumping, showing potential in laser application. Sun et al. reported 0D perovskite lasers based on pure phase Cs_4_PbBr_6_ microcrystals, which exhibits single‐ and multimode lasing resonance upon optical pumping with picosecond laser pulses.^[^ ^139^ ^]^ The PL spectra of Cs_4_PbBr_6_ microcrystal was narrowed with the increase of pump fluence, as shown in Figure 12e, indicating the occurrence of lasing resonance. The number of lasing modes can be tuned by adjusting the particle size. In addition, Cs_4_PbBr_6_ microcrystal laser shows much higher photostability than 3D CsPbBr_3_ based one, because the optically active defects can be efficiently protected by Cs_4_PbBr_6_ microcrystal as a matrix. Furthermore, they used the Cs_4_PbBr_6_ microcrystal laser as illumination sources to achieve high‐quality spectral‐free optical imaging due to the low spatial coherence of laser. Corresponding optical setup for speckle‐free imaging is shown in Figure 12f.

## 5 Conclusions and Outlook

0D perovskites have received widespread attention in recent years, due to their unique properties resulting from the peculiar structure, such as large exciton binding energy, strong quantum confinement effect, and excellent stability. Here, we comprehensively reviewed the various properties of 0D perovskites, including crystal and electronic structure, PL mechanism and regulation, and some special optoelectronic properties for 0D Cs_4_PbBr_6_. The PL of 0D perovskites is commonly from STEs, and their PL performance can be further optimized via chemical compositions and temperature regulation. However, the PL mechanism of 0D Cs_4_PbBr_6_ remains controversial and need further investigation. In addition, we summarized the synthesis methods of 0D perovskites for single crystals, colloidal NCs and thin films, and their applications in LEDs, solar cells, photodetectors, X‐ray detectors, scintillators, LSCs, electrochemical capacitors, and lasers. Although 0D perovskites have shown great potential in various applications, the related research is still in infancy and further study is needed to improve the devices performance.

It is noted that most of 0D perovskites are lead‐free perovskites, which is desirable for environmentally friendly alternatives and future development trend.^[^ ^140^ ^]^ We believe that 0D perovskites will move a significant step forward toward practical applications. However, most investigations on 0D perovskites are focused on the stage of materials development, and several challenges need to be addressed in the future to further promote the development and applications of 0D perovskites.

1) The design principle of 0D perovskites is still unclear. Although 0D perovskites containing various metal halide octahedra and clusters in various states have been developed, most of them can only be synthesized by trial and error. Therefore, it is necessary to establish rational synthetic control to prepare these materials. Since the optical and electrical properties of 0D perovskites are largely affected by their isolated metal halide octahedra and clusters, it is meaningful to achieve synthetic control for the size of metal halide octahedra and clusters and the distance between them in 0D perovskites. To realize such accurate control, developing computational methods to predict the forming process of 0D perovskites may be helpful.
2) Although many photophysical and electronic properties of 0D perovskites can be explained by the established theories and computational studies, there are remain challenges to fully understand the photophysical dynamics, especially on the excitonic behaviors on excited states. In addition, there is still much controversy on the PL mechanism of some 0D perovskites, especially on Cs_4_PbBr_6_. So, the further research to determine the origin of luminescence and reveal the exciton delocalization, exciton localization, and self‐trapping is necessary. To clarify this puzzle, more sophisticated techniques are required. For instance, taking advantage of in situ TEM to identify the origin of luminance on the single‐particle level, and using ultrafast spectroscopies to study the excited‐state dynamics.
3) The large bandgaps, defects, and relatively low PL QY of 0D perovskites have hindered their applications in optoelectronic devices. Therefore, some strategies should be adopted to modulate the bandgaps and optical properties of 0D perovskites for their better application in optoelectronic devices. A‐site/B‐site cation substitution has been widely reported as a potential method to realize the bandgaps modulation and PL optimization for 0D perovskites. However, some regulation methods via specific conditions have not been widely explored. For example, the pressure regulation may also help to the modulation of bandgaps and PL optimization. Defects have been claimed to induce luminance in some 0D perovskites,^[^ ^96^ ^]^ but the presence of defects has also been an issue for achieving high performance applications.^[^ ^32a^ ^]^ Many mature methods have been used to optimize 3D perovskites, such as ion doping, ligand engineering, and surface passivation, which can be attempted to improve the optical properties of 0D perovskites and further achieve high performance applications. In addition, the high exciton binding energy and poor charge‐carrier separation efficiency of 0D perovskites hinder their development in photovoltaic application, which are attributed to the strong quantum and dielectric confinements.^[^ ^32^, ^141^ ^]^ The quantum confinements in 0D perovskites can be reduced by enhancing the interinorganic cluster electronic coupling. For example, designing 0D perovskites with quantum‐well arrangement of inorganic clusters, which can result in the formation of delocalized excitons, enabling the facile separation of the excitons into free charge carriers.^[^ ^71b^ ^]^ The reduced dielectric confinements may be achieved by replacing the electronically inert A‐site organic cation with a polarizable cation,^[^ ^56^ ^]^ which can reduce the dielectric mismatch between the inorganic clusters and organic cation, thus leading to larger charge‐carrier mobility and lower exciton binding energy.
4) Investigations on the potential applications of 0D perovskites are still in a very early stage. Despite 0D perovskites have shown potential in various applications, most researches mainly focus on their applications in LEDs and solar cells. However, the current LEDs and solar cells based on 0D perovskites usually show undesirable device performances. So, reasonable device structure design and optimized 0D perovskites materials are both necessary to achieve high performance devices. We know that the large exciton binding energies enable room‐temperature polariton lasing that is potential to achieve low thresholds without the population inversion.^[^ ^142^ ^]^ 0D perovskites may be a potential material to achieve polariton lasing because they have large exciton binding energies, but there is no related report. So, the room‐temperature polariton lasing will be an important application direction of 0D perovskite to achieve low threshold laser. In addition, the research community may also turn their horizons to 0D perovskites for sensor application. Because the PL properties of some 0D perovskites can be changed under specific conditions (temperature, humidity, illumination, etc.), which makes them potential candidates for various types of sensors.

## Conflict of Interest

The authors declare no conflict of interest.
